# *In vitro* strategies for mimicking dynamic cell–ECM reciprocity in 3D culture models

**DOI:** 10.3389/fbioe.2023.1197075

**Published:** 2023-06-26

**Authors:** F. Urciuolo, G. Imparato, P. A. Netti

**Affiliations:** ^1^ Interdisciplinary Research Centre on Biomaterials (CRIB), University of Naples Federico II, Naples, Italy; ^2^ Department of Chemical Materials and Industrial Production (DICMAPI), University of Naples Federico II, Naples, Italy; ^3^ Center for Advanced Biomaterials for HealthCare@CRIB, Istituto Italiano di Tecnologia, Naples, Italy

**Keywords:** 3D tissue models, extracellular matrix, morphogeneis, dynamic reciprocity, cell–ECM interaction

## Abstract

The extracellular microenvironment regulates cell decisions through the accurate presentation at the cell surface of a complex array of biochemical and biophysical signals that are mediated by the structure and composition of the extracellular matrix (ECM). On the one hand, the cells actively remodel the ECM, which on the other hand affects cell functions. This cell–ECM dynamic reciprocity is central in regulating and controlling morphogenetic and histogenetic processes. Misregulation within the extracellular space can cause aberrant bidirectional interactions between cells and ECM, resulting in dysfunctional tissues and pathological states. Therefore, tissue engineering approaches, aiming at reproducing organs and tissues *in vitro*, should realistically recapitulate the native cell–microenvironment crosstalk that is central for the correct functionality of tissue-engineered constructs. In this review, we will describe the most updated bioengineering approaches to recapitulate the native cell microenvironment and reproduce functional tissues and organs *in vitro*. We have highlighted the limitations of the use of exogenous scaffolds in recapitulating the regulatory/instructive and signal repository role of the native cell microenvironment. By contrast, strategies to reproduce human tissues and organs by inducing cells to synthetize their own ECM acting as a provisional scaffold to control and guide further tissue development and maturation hold the potential to allow the engineering of fully functional histologically competent three-dimensional (3D) tissues.

## 1 Introduction

Tissue-engineered products are generally intended to regenerate, repair, or replace human tissue. However, these products have recently also been used as functional human tissue models in laboratory settings for the purpose of drug discovery, toxicity testing, and disease modeling. In this perspective, the goal is to replicate the complex microarchitecture and physiological functions of human tissues and create models that more accurately reflect human biology when compared to the traditional human experimental assay such as cell cultures or animal models (Ishida, 2018; Langhans, 2018). The major challenge is to recapitulate the complexity of the native cell and tissue microenvironment, which includes the composition and structure of the ECM, and the time and space presenting processes of biochemical and biophysical signaling molecules (Rozario and DeSimone, 2010; Thorne et al., 2015; Sainio and Järveläinen, 2020). When aiming for this, the structure and chemical nature of the scaffold material play a pivotal role and should control and guide the specific molecular or cellular events such as molecular and cellular recognition, morphogenesis, tissue remodeling, and cell repair by responding to changes in the biological environment or the transformation of cells from one state to another by adapting and synchronizing the time and space presentation of multiple arrays of biochemical and morpho-physical signals. However, attempts to integrate space and time signal presentation control within synthetic or semi-synthetic materials have often led to disappointing results due to the difficulty in replicating the sophisticated logic of signal presentation that is encoded within the native ECM (Rozario and DeSimone, 2010).

The ECM is a complex network of proteins and polysaccharides that surrounds and supports the cells in a tissue. Although formed by the same structural units (i.e., elastin, collagen, hyaluronan, proteoglycans, fibronectin, and laminin), the specific organization and amount of structural units of the ECM vary from organ to organ. Furthermore, in the same organ, spatial differences in the ECM can be observed. The dermis, for instance, presents two regions, the papillary and reticular dermis. They are produced by the same cells, the fibroblasts, and contain the same macromolecules that are differently organized, resulting in different final functions and properties (Zhao et al., 2019). The dermal ECM not only performs the function of structural support but also plays the key role in epidermal/dermal cross-talking which is responsible for hair follicle morphogenesis and cycling (Rozario and DeSimone, 2010). In the heart, the ECM provides mechanical support to the myocardial cells and helps maintain the structural integrity of the heart and regulates the contraction of the heart by providing a scaffold for the organization of the sarcomeres. Additionally, the ECM plays a role in the repair and regeneration of the heart after injury by the recruitment and proliferation of resident cardiac progenitor cells (Corda et al., 2000; Zhang et al., 2021a). In the lungs, the ECM provides structural support to the alveoli and bronchi and also helps maintain the specific mechanical properties of the lung tissue. The ECM also dictates lung development by providing the correct spatiotemporal signal presentation to guide the growth and branching of the lung epithelial cells (Range and Moser, 2012; Burgess et al., 2016; Burgstaller et al., 2017). Other than being site specific, the ECM is also status specific by changing its composition and signal presentation with aging, pathologies, and other extrinsic factors (e.g., diet, UV exposure, and pollutants; Selman and Pardo, 2021; Rybinski et al., 2014). Any pathological variation in the ECM properties leads to organ dysfunction. Fibrotic tissues, for instance, are composed of the same macromolecules as that of healthy tissues, but the aberrant growth of collagen when compared to other ECM macromolecules, its stiffening, and the variation in fiber organization compromise the functions of the organs (Zhao et al., 2019; Burgess et al., 2016; Burgstaller et al., 2017). These pieces of evidence highlight that cells cannot be decoupled by their own ECM, and when cells are seeded in an exogenous/synthetic context, dysfunctional tissues are obtained. The awareness of the strict relationship between a cell and its specific microenvironment represents a paradigm shift in scaffold designing, highlighting the necessity to fabricate functionalized biomaterials capable of replicating the regulatory cell function of the native ECMs. In this review, we discuss the different approaches proposed to mimic the ECMs *in vitro*, which include the use of natural and synthetic ECM mimetic (Saska et al., 2021; Assunção et al., 2020; Gjorevski et al., 2014; Parenteau-Bareil et al., 2010; Hutmacher et al., 2004; Lutolf and Hubbell, 2005; Swinehart and Badylak, 2016; Saludas et al., 2017; Abbasian et al., 2019; Guddati et al., 2019; Hosoyama et al., 2019; Del Prado-audelo et al., 2020) and the challenges that have to be still overcome to realize a perfect replica of the ECM for tissue engineering applications (Netti, 2019). In addition, the functionalization of synthetic biomaterials is discussed together with the use of organ-derived ECMs and bio-inks for 3D printing. We pay particular attention to tissue engineering strategies in which somatic cells are induced to produce their own ECM (Roy et al., 2020; Urciuolo et al., 2016), showing that the resulting bioengineered organs and tissues can replicate *in vitro*, and the relevant biological processes that are strictly related to the cell–ECM interaction (De Gregorio et al., 2017; Imparato et al., 2017; Lombardi et al., 2017; Casale et al., 2018; Mazio et al., 2018; De Gregorio et al., 2020).

## 2 Cell–ECM bidirectional reciprocity: implication in morphogenesis and disease

The ECM is a macromolecular network that provides structural support, defines tissue architecture, and elicits signals relying on the status of the mechanical environment to adherent cells (Miller et al., 2020).

From the discovery of integrins (and other ECM receptors) in the mid-1980s, the past concept of the ECM being a “passive” scaffold holding cells and tissue in place has been overcome, and the ECM’s regulatory role on cell functional states in normal physiology and homeostasis, disease progression, and development has been widely recognized (Rozario and DeSimone, 2010; Dede Eren et al., 2022). Integrins are a family of heterodimeric receptors composed of an α- and β-subunit that mediate cell adhesion to a number of ECM proteins. Upon binding with an ECM ligand, integrins transmit signals that activate a number of intracellular signaling pathways (Faralli et al., 2022). Integrins engage ECM components with their extracellular domains and cytoskeletal and signaling proteins via their cytoplasmic tails. Through these connections, integrins provide a mechanical link between the ECM and cytoskeleton, allowing cells to sense and respond to mechanical cues from the ECM (Kanchanawong and Calderwood, 2023). The main class of ECM’s macromolecules involved in the regulation of cell signaling includes collagens, proteoglycans, elastin, and glycoproteins such as fibronectin and laminin. The collagens, which are the most abundant ECM proteins, are responsible to provide structural support for tissues (Sainio and Järveläinen, 2020). Proteoglycans have both structural and biological roles as they are responsible for the mechanical resistance to compression and hydration of the tissues and serve to trap growth factors (GFs) in the ECM.

Elastin and fibrillin are the main components of elastic fibers and are both critically important in the development and homeostasis of elastic tissues (Sainio and Järveläinen, 2020; Karamanos et al., 2021). In particular, fibrillin microfibrils mediate cell signaling via integrin and syndecan receptors, and microfibrils sequester the transforming growth factor β (TGF-β) family GFs within the matrix to provide a tissue store which is critical for homeostasis and remodeling (Godwin et al., 2019). Fibronectin and laminin are non-collagenous ECM glycoproteins, of which the former is an important regulator in the cell–ECM signaling process, while the latter is the most present component of the basement membrane and can modulate cell adhesion, differentiation, and migration. A variety of other molecules are present in the ECM, such as cytokines, chemokines, metalloproteinases (MMPs), and their inhibitors. All these biochemical signals together with the biomechanical signals (stretching, shear stress, stiffness, and surface topography) are involved in the modulation of cellular phenotype, shape, and functions (Dede Eren et al., 2022). Cells in turn constantly deposit, degrade, or modify the ECM to carry out their functions such as growth, apoptosis, and differentiation (Sainio and Järveläinen, 2020). The continuous and dynamic interaction between cells and their surrounding environment affects biomechanical and biochemical properties of the ECM and cell function through activation of signal transduction pathways that regulate gene and protein expressions (Dede Eren et al., 2022). The entirety of these bidirectional interactions between cells and their surrounding ECM is referred to as cell–ECM dynamic reciprocity and represents the key driver of most important biological processes such as development and disease, as well as reproduction and embryogenesis (Thorne et al., 2015; Turley et al., 1991; Knudson and Peterson, 2004; Burgess et al., 2019; Nemec and Kilian, 2021).

In the following section, we provide a sampling of ECM functions in pathophysiological events occurring in the human body and highlight the diversity of mechanisms that depend upon the actions of matrix molecules and their cellular receptors (Range and Moser, 2012).

### 2.1 Branching morphogenesis

The development of branched organs is an interesting example of the multiple roles played by the ECM in morphogenesis. The branching involves the invasion of epithelial buds and tubes into the surrounding embryonic mesenchyme rich in ECM. Several matrix molecules such as glycosaminoglycans (GAGs), collagens, and many other glycoproteins are involved as regulators of hair follicle, mammary gland, salivary gland, kidney, gut, and lung development. The branching units are surrounded by microenvironments of the ECM that change in composition and spatial distribution over time (Rozario and DeSimone, 2010). This continuous remodeling of the ECM within a changing microenvironment supplies the morphogenic cues to control cell survival, proliferation, migration, polarization, and differentiation, while the cell’s cytoskeleton mediates the extra- to intracellular crosstalk that occurs between the nucleus and microenvironment (Fata et al., 2004; Nelson et al., 2006; Coates et al., 2011a).

The mammary glands are a unique branched organ in which most of the branching morphogenesis are required to develop the ductal tree, which occurs postnatally during puberty. Therefore, they represent a deeply investigated model to understand how the ECM remodeling contributes to tissue morphogenesis and functional differentiation (Coates et al., 2011a). The mammary gland presents many cell types such as fibroblasts, adipocytes, and epithelial cells. The latter, embedded in an interstitial ECM, are present as luminal epithelial and myoepithelial cells and are both appointed to form the branching network of ducts terminating in small lobuli named acini (Thorne et al., 2015). The ECM expression patterning along the branching structures is strongly heterogenous, where ECMs rich in collagen IV, laminin I, and laminin 5 are found around the acini, while collagen I is expressed along the mammary ducts (Thorne et al., 2015; Fata et al., 2004; Silberstein and Daniel, 1984). The heterogenous ECM expression patterning is the result of the ECM constant assembly and degradation and provides the correct spatiotemporal cue presentation necessary to guide the cells toward the different stages of mammary gland development and functioning (branching, alveogenesis, lactation, and involution) (Coates et al., 2011a; Kilmer, 2010). Among the ECM’s components, the fibronectin plays a crucial role in gland development; indeed, it increases appreciably during ductal morphogenesis as do expressions of the fibronectin receptor α5β1 integrin in the myoepithelial cells (Kilmer, 2010). The loss of fibronectin expression results in dysfunctional gland development (Liu et al., 2010; Thorne et al., 2015). Spatiotemporal expressions of MMPs is necessary for the remodeling of the external environment; MMP-2 plays a role in the initial invasion of epithelial cells into the stromal fat pad while MMP-3 promotes branching (Fata et al., 2004; Wiseman et al., 2003).

The interactions between the mammary cells and the ECM have been extensively investigated in 3D cultures aiming at modeling mammary gland morphogenesis. Evidence from experimental studies have shown that fibronectin expression decreases during acinar morphogenesis as cells polarize and form a lumen. In addition, the supplement of exogenous fibronectin increases cell proliferation and colony size, suggesting the role of this ECM component in coordinating epithelial cell growth during mammary gland development (Hynes et al., 1979). During lactation, myoepithelial and luminal epithelial cells secrete milk into the lumen of the acini. Laminin-111 is the basement membrane component secreted by the myoepithelial cells that trigger the polarization of luminal epithelial cells (Kleinman et al., 1986). The latter cultured *in vitro* in 3D laminin-rich ECM can establish apical–basal polarity and express milk proteins in response to lactogenic hormones even in the absence of myoepithelial cells. On the contrary, if cultured in collagen gels lacking laminin, the cells display reversed polarity and lose mammary-specific gene expression (Coates et al., 2011a; Coates et al., 2011b), confirming the critical role of the ECM in guiding mammary-specific function (Coates et al., 2011b). These insights demonstrate that the ECM can direct tissue polarity and morphogenesis and even affect gene expression and nuclear remodeling, providing an unequivocal proof that ECM–cell interactions are necessary for mammary gland development and functioning, substantiating a role for dynamic reciprocity in the breast (Thorne et al., 2015).

### 2.2 Alterations in ECM during organ disease

In physiological conditions, dynamic reciprocity works to guarantee the homeostasis in human tissue, but any defect in the mechanochemical signaling network can trigger tumorigenesis. In its natural state, the mesenchymal–stromal cells establish a tightly controlled environment which guarantees a tumor-repressive homeostatic equilibrium regulated by local fibroblastic cells (Alexander and Cukierman, 2016). However, critical pathological events such as chronic inflammation and cancer can forbid the restoration of an innate mesenchymal homeostatic state (Rybinski et al., 2014; Alexander and Cukierman, 2016; Kunz-Schughart and Knuechel, 2002). When this situation occurs, all the stromal components undergo modification evolving toward a new equilibrium that preserves the pathological condition (Alexander and Cukierman, 2016); Hu and Polyak, 2008). This homeostatic stromal change is evident in most carcinomas in which the stroma compartment is characterized by a fibrosis-like reaction called desmoplasia. The desmoplastic stroma presents activated fibroblastic cells (myofibroblasts) that are responsible for specific modifications in the ECM architecture and composition, such as increased type I collagen deposition and, in contrast to innate stromal features, an anisotropic collagen network organization (Kunz-Schughart and Knuechel, 2002). In turn, the ECM affects cellular activity via changes in the cytoskeleton and subsequently drives the expression and secretion of the matrix remodeling molecules, such as collagen cross-linkers and MMPs. This ‘mechanotransduction’ is modulated by the integrins, the bi-directional ECM–cell receptors acting as a link that enables the transmission of physical and chemical cues from the extracellular environment to the nucleus (Jahed et al., 2014). In the cancer microenvironment, the fibrotic and desmoplastic stromal environment represents the fuel that sustains myofibroblastic activation (Alexander and Cukierman, 2016; Webber et al., 2015). Indeed, as cells become contractile, the mechanical strain increases stretching the ECM fibers that in turn make the ligands accessible to integrins (Kubow et al., 2009). Taken together, the observations report and highlight that the cell matrix bidirectional reciprocity occurring in cancer is responsible for two dynamic processes: the stromal myofibroblastic-imposed effect that is responsible for remodeling the ECM landscape and the ECM-imposed cellular influence (Alexander and Cukierman, 2016; Malik et al., 2015). During both processes, biochemical signaling cascades are regulated through cell–ECM receptors that stimulate intracellular changes mediated by cytoskeletal reorganization (Thorne et al., 2015; Alexander and Cukierman, 2016). Cancer is not the only disease where the dynamic reciprocity of the cell–ECM regulates the initiation and progression of the pathological status, recently it has emerged that the cell–ECM interchange plays a crucial role in the initiation and evolution of chronic lung diseases (Thorne et al., 2015; Burgess et al., 2016; Burgstaller et al., 2017). Due to the increase in knowledge about the alterations in the profiles of ECM proteins in diseased lung tissues, recently there has been a growing need to understand the functional significance of these changes and how the composition of the ECM contributes to disease pathology in airways. It is well known that the asthmatic airway is characterized by alterations in the epithelial cells, smooth muscle cells, blood vessels, and in the ECM structure, and now, the correlation between the airway structure and other lung pathologies [chronic obstructive pulmonary disease (COPD) and interstitial lung fibrosis] is under investigation (Larsen et al., 2015; LaPolt and Lu, 2001). The airway smooth muscle cells (ASMs) produce and secrete several ECM proteins and MMPs, influencing their surrounding microenvironment that in turn affects the proliferative, migratory, and synthetic responses of the ASM cells. TGF-β, a pro-fibrogenic growth factor that has been implicated in airway remodeling in asthma and other fibrotic lung diseases, is anchored in the ECM, providing a reservoir of this GF that can be released on demand. Among its many functions, TGF-β regulates the deposition of ECM proteins by ASM cells (Johnson et al., 2006; Xie et al., 2007). Therefore, the TGF-β activation influences the balance between ECM production and degradation. The disruption of the ECM may be the key driver for the induction of the fibrotic process. Fibroblasts are also target cells for ECM-modulated effects in lung disease. The asthmatic-derived elongated fibroblasts produce higher amounts of biglycan, decorin, and versican and migrate twice as far as the fibroblasts originating from bronchial biopsies from the same patients, suggesting that the altered ECM profile contributes to the migratory phenotype of the elongated fibroblasts (Larsen et al., 2004). Investigations on the changes in the ECM in COPD patients have also been carried out. It has been shown that the deposition of ECM proteins in COPD patients’ lung tissues is mainly driven by fibroblasts, which produce a versican-rich ECM that inhibits the formation of elastin fibers (LaPolt and Lu, 2001), (Hallgren et al., 2010). Furthermore, the structure of the ECM in patients with idiopathic pulmonary fibrosis is different from that of healthy patients. Indeed, fibroblasts derived from patients with pulmonary fibrosis produce high levels of hyaluronan and decorin and present with lower proliferative rates than those with low levels of these ECM proteins (Westergren-Thorsson et al., 2004). The fibrotic deposit in the lung tissue seems to be both a cause and consequence of fibroblast activation. In addition, the ECM in fibrotic lungs (Fernandez and Eickelberg, 2012) is not only altered in composition but also more rigid than it is in non-diseased lung tissue (Booth et al., 2012). All together, these data demonstrate that the ECM in the airway tissues of patients with fibrotic lung diseases can dictate cellular behaviors and add to or modulate disease pathology.

## 3 Bioengineered approaches to mimic cell microenvironment complexity

**FIGURE 1:**
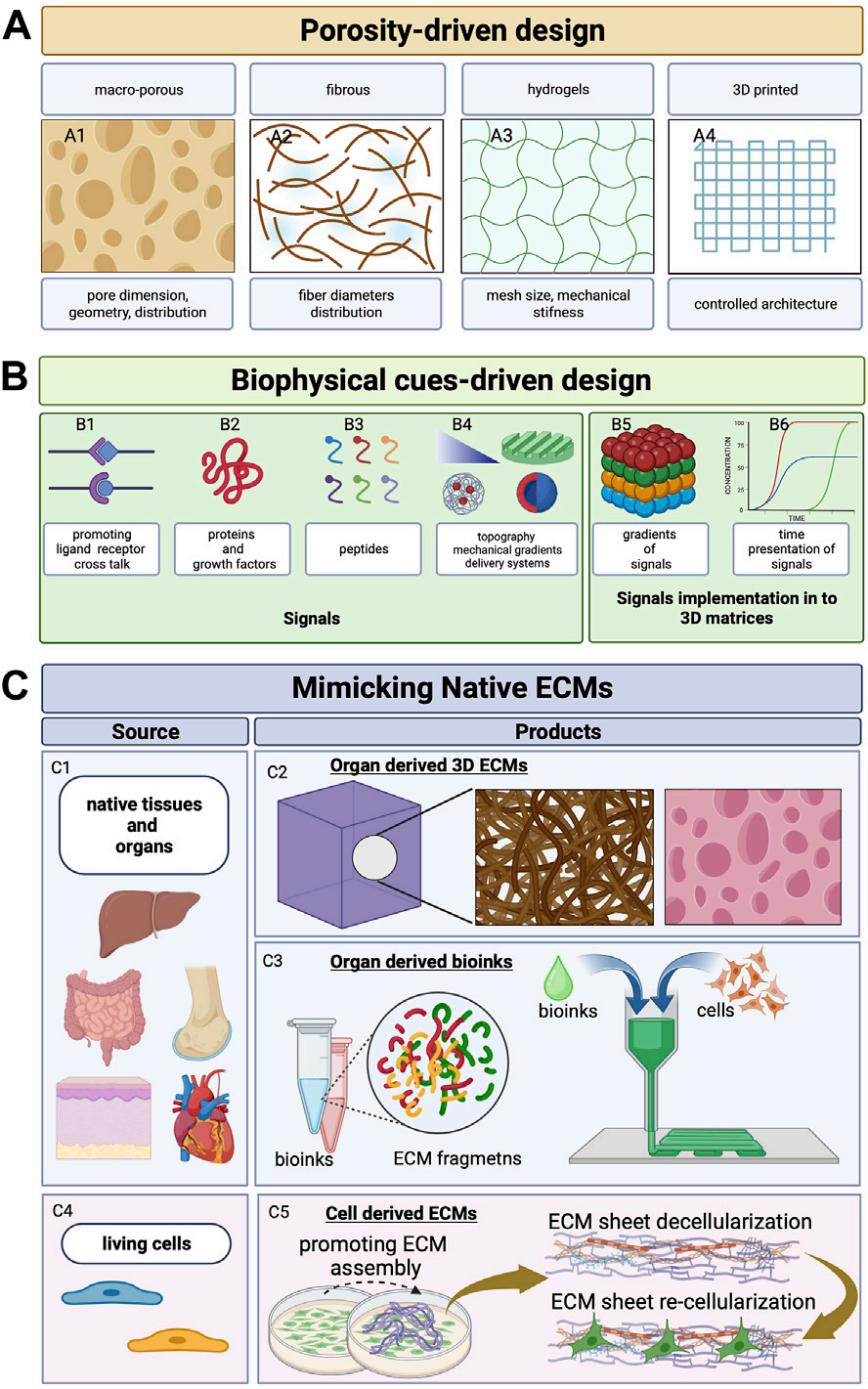
Scaffold design evolution. **(A)** Schematic representation of the evolution of the scaffold design concept. Porous 3D architecture can be formed as macro-porous matrices (A1; Eltom et al., 2019; Loh and Choong, 2013), fibrous matrices that are woven/non-woven/knitted (A2; Braghirolli et al., 2014), hydrogels with controlled swelling and mechanical stiffness (A3; Lee and Mooney, 2001; Drury and Mooney, 2003; Braziulis et al., 2012; Hoffman, 2012), and 3D printing (A4; Patra and Young, 2016). **(B)** Signals that can be encoded in 3D architecture to mimic the ECM’s role—ligands (B1), proteins (B2), peptides (B3), mechanical gradients (triangle in B4), patterns (patterned surface in B4), and delivery systems for controlled release of growth factors that can be arranged as spatial gradients (B5) with programmed and time-controlled release (B6) (Lutolf and Hubbell, 2005; Del Prado-audelo et al., 2020; Kim et al., 2017; Lutolf et al., 2003; Patra and Young, 2016; Das and Noh, 2018). **(C)** Strategies to mimic native ECM by using decellularized native tissue and native organs as source (C1) to obtain 3D porous structures (organ-derived 3D ECMs, C2; Rajab et al., 2020; Mazza et al., 2015) or organ-specific bio-inks for bioprinting applications (organ-derived bioinks, C3; Choudhury et al., 2018). By stimulating stromal cells (i.e., fibroblasts or MSCs, C4) or by means of macromolecular crowding or ascorbate, site-specific ECM sheets are obtained (C5). Such ECMs populated by cells are then (i) used as living cell sheets (Roy et al., 2020) or (ii) decellularized and re-seeded with other cell types (Yong et al., 2020). Created with BioRender.com.

Tissue engineering approaches rely on the use of biomaterials acting as ECM surrogates to support cell migration, survival, proliferation, and biosynthetic activity. To accomplish the abovementioned properties of the native extracellular space, different biomaterials of either natural or synthetic origin arranged in the form of macroporous materials, fibrillar network, and swollen hydrogels have been employed (Assunção et al., 2020; Hutmacher et al., 2004; Eltom et al., 2019). The design criteria for the scaffolds acting as ECM surrogates involve the definition of the internal architecture from the nano- to micrometric levels, surface properties (such as roughness, wettability, and chemistry), and topographical and mechanical features. Such design criteria can be subdivided into porosity-driven design and biophysical cue–driven design (Figures 1A, B). The former involves the definition of suitable porosity, interconnectivity, pore shape, and dimension, while the latter involves the use of bio-inspired molecular signals with controlled spatiotemporal presentation to the cell receptors: the mechanical and non-topographical cues.

### 3.1 Porosity-driven design

Scaffolds with controlled porosity and degree of interconnectivity can be obtained by using different processing approaches such as (i) bulk processing (gas foaming, solvent casting/porogen leaching, thermally or chemically induced phase separation, and freeze drying) (Yadav et al., 2021), (ii) fiber formation and successive assembly into 3D fibrous structures (e.g., electrospinning, fiber bonding, and textile-derived techniques) (Rnjak-Kovacina et al., 2011; Wubneh et al., 2018), and (iii) polymer network cross-linking to form hydrogels (Saludas et al., 2017; Braziulis et al., 2012; Yang et al., 2017). Process variables such as porogen concentration, temperature, pressure, electric field, polymer concentration, and degree of cross-linking can be used to modulate scaffold properties which may ultimately trigger and modulate specific cellular functions. Pore size, distribution, and dimensions have been shown to affect both cell proliferation and differentiation (Loh and Choong, 2013; Rnjak-Kovacina et al., 2011). Takahashi and Tabata (2004) showed that fiber diameter and porosity of 3D non-woven matrix fabricated using polyethylene terephthalate (PET) fibers affected osteogenic differentiation and proliferation of rat mesenchymal stem cells (MSCs) . By using 3D porous scaffolds based on silk fibroin, it was demonstrated that pore sizes ranging from 200 to 250 μm and porosity of approximately 86% enabled better proliferation of foreskin fibroblasts (Mandal and Kundu, 2009). Other studies have reported that the average pore size should be approximately 35 μm for stimulating vascularization, between 20 and 125 μm for enhancing fibroblasts in growth and skin regeneration, and between 100 and 350 μm for bone regeneration (Netti, 2019). Other than cell proliferation, migration, and differentiation, porosity and pore size have been shown to influence cellular biosynthetic activity. In studies concerning cartilage tissue engineering, it has been shown that chondrocytes seeded in a porous gelatin scaffold displays preferential proliferation and ECM production for scaffolds with pore sizes between 250 and 500 μm (Lien et al., 2009). In addition, pore size and porosity have been shown to influence MSC differentiation (Ferlin et al., 2016; Matsiko et al., 2015), adipogenesis (Guneta et al., 2016), bone tissue regeneration (Kuboki et al., 2001), hepatogenesis, skin regeneration, and smooth muscle differentiation (Loh and Choong, 2013). Further advancements in scaffold manufacturing have been obtained with the advent of additive manufacturing techniques (e.g., 3D printing, selective laser sintering, stereo lithography, and fused deposition modeling) and microfabrication approaches. These methods have enabled better control at the micro- and macroscales over pore dimension, shape, orientation, spatial distribution, and porosity (Guddati et al., 2019; Taniguchi et al., 2016). The copious research conducted so far have demonstrated that both pore size and shape can modulate specific mechano-transduction pathways. The cells seeded onto pore surfaces have the capacity to sense geometrical features by experiencing mechanical stresses that induce cytoskeleton rearrangement triggering nuclear deformation that ultimately affect gene expression. Pereira et al. (2020) demonstrated a shape-dependent behavior (square vs. rectangle) of myoblast cells (C2C12 line) by demonstrating that square pores led to higher nuclear localization for histone lysine methyltransferase—SMYD3, histone lysine trimethylation, and YAP/TAZ than did the rectangle pattern. Another study used methacrylate hyaluronic acid (MeHA) to encapsulate human-derived MSCs in pores having different geometries such as triangular, cylinder, cuboid, and cube and showed that triangular and cuboid pores induced higher nuclear YAP/TAZ localization (Bao et al., 2017). The effect of pore shape/dimension on cell fate can be also related to the curvature of the pores. Smaller pores have steeper curvature than large pores, inducing different cytoskeleton organization that ultimately controls cell differentiation pathways. Finally, the shape, dimension, and curvature of the pores can control ell fate by modulating the stress fibers and F-actin polymerization, focal adhesion formation and cell tension, nuclear functions, and mRNA concentration in cells (Bao et al., 2017; Swanson et al., 2022). Once an optimal pore shape and dimension is established, the other parameters can have effect on cell behavior such as the dynamic culture conditions (Martin et al., 2004; Urciuolo et al., 2011). In perfusion bioreactors, given the shape and distribution of pores, the perfusion flow rate can modulate cell behavior due to fluid dynamic conditions. It has been shown that MSCs seeded in poly-caprolactone porous scaffolds experienced bone differentiation when the flow rate could establish a specific shear stress distribution at the cell surface (Guarino et al., 2012). In particular, a shear stress distribution at the cell/fluid interface falling in the range of 10^−3^–1 Pa maximized the expression of osteopontin, alkaline phosphate, and osteocalcin when compared with other fluid dynamic conditions.

### 3.2 Biophysical cue-driven design

Besides pore size, shape, interconnectivity, and spatial arrangement, many other important cues have to be implemented and opportunely modulated in the 3D scaffolds if one wishes to mimic the dynamic and instructive role of native extracellular space as close as possible. Therefore, cell instructive materials hosting molecular, topographical, mechanical, and morphogenetic cues have emerged as a new class of advanced biomaterials for tissue engineering applications (Ventre et al., 2012; Custódio et al., 2014).

*Molecular cues* that are provided to cells arranged in 3D contexts can be divided into ECM-binding proteins (collagen, elastin, adhesive glycoproteins, gelatin, vitronectin, fibronectin, and laminin), ECM-remodeling proteins, and GFs. The importance of ECM-binding proteins in *in vitro* cell cultures to promote their adhesion is widely recognized. Adsorption of such proteins on biomaterial surfaces to support cell adhesion is a common practice in tissue engineering applications. In addition, different biomaterial scaffolds are produced in the form of hydrogels or sponges made up of ECM proteins (Del Prado-audelo et al., 2020). Type I collagen and fibrin hydrogels with tunable local stiffness and fiber diameters have been used to modulate cell mechano-sensing properties and migration (Münster et al., 2013). Furthermore, collagen-based scaffold and its composites (i.e., in combination with other ECM proteins) have been used in different tissue engineering applications comprising regeneration of tendons, skin, vascular grafts, heart valves, and dental/bone applications (Del Prado-audelo et al., 2020; Parenteau-Bareil et al., 2010). Besides ECM proteins, GFs represent important signaling molecules that modulate cellular activities. The GFs can be sequestered by the ECM for their presentation to cell receptors to stimulate cell migration, growth, proliferation, differentiation, and gene expression. They play important roles in wound healing, tissue regeneration, and immune regulation. Among the many known GFs, bone morphogenetic proteins (BMPs), insulin-like GF-1 (IGF-1), TGF-β, basic fibroblast GF-2 (FGF-2), platelet-derived GF (PDGF), and vascular endothelial GF (VEGF) are the most used for tissue engineering applications (Klimek and Ginalska, 2020). Both ECM proteins and GFs if correctly presented in terms of dose–spatial–temporal presentation can correctly stimulate a constructive cell response in different mechanisms such as wound repair and tissue integration. To mimic the native spatial and temporal signal presentation, GFs have been coupled with suitable biomaterials acting as GF delivery systems to allow their preservation and activity by enabling their sustained and on-demand release (Klimek and Ginalska, 2020; Subbiah and Guldberg, 2019). The coupling of GFs with suitable biomaterials can be obtained by entrapping them in porous/non-porous nanotubes, fibers, particles, capsules, spheres, or hydrogel matrices. In the landscape of GF release for tissue regeneration applications, pH-, redox-, and temperature-sensitive GF delivery strategies have been largely employed. Kim et al. (2017) conjugated the epidermal growth factor (EGF) to polymer fiber patches by means of MMP cleavage sequences. Once exposed to over-expressing MMP environments, the genetically engineered EGF was rapidly released favoring the migration of keratinocytes in wound-healing models. pH-sensitive alginate/CaCO_3_ composite microparticles of approximately 400 µm in diameter, loaded with bFGF realized with microfluidic techniques, improved the antacid ability of the microparticles and reduced the initial burst release. Slow and sustained release of bFGF was achieved, and significant keratinocyte proliferation and migration rates both *in vivo* and *in vitro* were observed (Shi et al., 2019). Other relevant applications of stimuli-responsive systems can be found in the field of bone and cartilage regeneration, heart and skeletal muscle repair, nerve growth, and vascularization (Qu et al., 2020). With these strategies, different delivery kinetics can be obtained such as burst, sustained, delayed, and pulse-like. The challenge in GF release platforms is represented by the loading of different GFs in the same matrix in order to promote multiple delivery with the aim to increase their synergic effect and control their spatiotemporal on-demand presentation (Subbiah and Guldberg, 2019). Spatiotemporal delivery has been improved by the recent advances in additive manufacturing: micro- or nanoparticles loaded with biomolecules can be deposited along precise 3D architectures inside the scaffolding material (Fahimipour et al., 2017; Zhu et al., 2018). Such carriers act as protecting materials against GF degradation, and the release kinetic can also be modulated by adjusting material properties. Furthermore, their spatial localization can be controlled during manufacturing, with the final result having a spatial and temporal controlled release (Salerno and Netti, 2021; Tarafder et al., 2016; Wen et al., 2019; Subbiah et al., 2020). Whole proteins and GFs can be difficult to manipulate during the bioconjugation steps. This leads to the identification of amino acid sequences that elicit cell responses analogous to those provided by the proteins. Such peptides bring the advantages of being more stable than whole proteins and are easier to manipulate and synthesize.

The RGD (Arg-Gly-Asp) sequence is the most important modifier of biomaterials as it has been recognized to be a pro-adhesive motif, via integrin binding, found mainly in collagen, gelatin, fibronectin, and laminins (Pountos et al., 2016; Krishna and Kiick, 2010; Gjorevski et al., 2014). Other than to control cell adhesion and mechanotransduction, the integrin/RGD binding has been demonstrated to have an effect on oxygen consumption kinetics. Guaccio et al. (2008) clearly reported that given a cell line and scaffold porosity, the RGD concentration affected the oxygen consumption kinetic parameter. In particular, the higher the RGD concentration, the lower was the oxygen request, indicating that when the cells were engaged in interactions with the ECM peptides, the metabolic request was lower. Different amino acid sequences found in both the ECM and GFs that are commonly used in tissue engineering applications are summarized in Table 1 and described in detail elsewhere (Hosoyama et al., 2019; Asghari Sana et al., 2017; Zachman et al., 2013; Lin et al., 2010; Kim et al., 2012; Zhang et al., 2016; Stahl et al., 2014). Other molecules include peptides that are either ECM- or GF-derived and belong to the category of self-assembly peptides (SAPs) (Lutolf and Hubbell, 2005). Moreover, the extracellular environment found in native tissues is subjected to continuous proteolytic activity via cleavage of ECM fragments sensitive to remodeling enzymes, such as MMPs and serine proteases and hyaluronidases, to allow ECM turnover and cell migration. Such proteolytic-mediated migration is part of the cell–ECM reciprocity mechanism. To replicate such dynamic interplay, several synthetic biomaterials (e.g., PEG hydrogels) have been functionalized with molecular motifs that are sensitive to proteases (Lutolf and Hubbell, 2005). The simultaneous incorporation of adhesion ligands and protease-sensitive motifs into polyethylene glycol (PEG) networks has been demonstrated to enable integrin-dependent proteolytic 3D migration of fibroblasts and endothelial cells. Such materials have enabled a fine control over cell migration by acting on different properties of the 3D matrix: physicochemical characteristics (extent of cross-link and polymer concentration), adhesion ligand density, or proteolytic sensitivity of cysteine-containing peptides such as GCRRG or GRCRG (where R is arginine, G is glycine, and C is cysteine) (Lut et al., 2001; Halstenberg et al., 2002; Lutolf et al., 2003).

**TABLE 1:** ECM- and GF-derived peptides: examples, functions, and applications.

ECM-derived peptides	Applications and function
• DGEA (Asp-Gly-Glu-Ala)	Involved in integrin signaling that can promote cell adhesion, proliferation, and differentiation (Hosoyama et al., 2019)
• GFOGER (Gly-Phe-Hyp-Gly-glu-arg)	
• GFPGER (Gly-Phe-Pro-Gly-Glu-Arg)	
• PepGen P-15 (P-15): GTPGPQGIAGQRGVV (Gly-Thr-Pro-Gly-Pro-Gln-Gly-Ile-Ala-Gly-Gln-Arg-Gly-Val-Val)	Class of the pro-adhesive collagen-derived peptide. It is known to stimulate osteoblast adhesion and proliferation (Hennessy et al., 2009)
• PHSRN (Pro-His-Ser-Arg-Asn)	Fibronectin-derived sequences. It been demonstrated that their use is to enhance adhesion and proliferation of fibroblasts, MSCs, and endothelial cells (Hosoyama et al., 2019; Asghari Sana et al., 2017)
• REDV (Arg-Glu-Asp-Val)	
• LDV (Leu-Asp-Val)	
• KQAGDV (Lys-Gln-Ala-Gly-Asp-Val)	
• C16: KAFDITYVRLKF (Lys-Ala-Phe-Asp-Ile-Thr-Tyr-Val-Arg-Leu-Lys-Phe)	Other than pro-adhesive sequences, peptides from laminins have been found to have the pro-angiogenic feature. C16 enhances endothelial cell migration, adhesion, and proliferation *in vitro* and can support angiogenesis *in vivo* (Zachman et al., 2013)
**GF-derived peptides**	
• P17: IVAPPGYHAFYCHGECP (Ile-Val-Ala-Pro-Pro-Gly-Tyr-His-Ala-Phe-Tyr-Cys-His-Gly-Glu-Cys-Pro)-	BMP-derived peptides. It has been shown to enhance viability of bone marrow stem cells and stimulate osteogenic differentiation and bone regeneration in combination with natural and synthetic scaffolds *in vivo* and *in vitro* (Lin et al., 2010; Kim et al., 2012; Zhang et al., 2016)
• P24: KIPKASSVPTELSAISTLYLSGGC (Lys-Ile-Pro-Lys-Ala-Ser-Ser-Val-Pro-Thr-Glu-Leu-Ser-Ala-Ile-Ser-Thr-Leu-Tyr-Leu-Ser-Gly-Gly-Cys)-	
• BFP1: GQGFSYPYKAVFSTQ (Gly-Gln-Gly-Phe-Ser-Tyr-Pro-Tyr-Lys-Ala-Val-Phe-Ser-Thr-Gln)-	
• QK: KLTWQELYQLKYKGI (Lys-Leu-Thr-Trp-Gln-Glu-Leu-Tyr-Gln-Leu-Lys-Tyr-Lys-Gly-Ile)	Amino acid sequences that mimic the VEGF functions by eliciting endothelial cell migrations, proliferation, and angiogenesis (Stahl et al., 2014)

*Nanopatterns and mechanical cues* represent other important sets of signals useful for bioengineering the cell microenvironment and to control cell fate. The advent of even more sophisticated microfabrication techniques such as photolithography, electron beam lithography, microcontact printing, microfluidics, and two photons has provided the possibility to fabricate biomaterials with specific nanometric features. Nanopatterning in the form of grooves and channels, pillars, wells and pits, and molecular motifs can be used to modulate adhesion, alignment, migration, and differentiation of the cells. Gradients of such signals can be obtained by spatially varying the pattern density. Patterning can be used to induce cell alignment or to orchestrate precise spatial distribution to mimic specific tissue architectures. Patterned collagen gel has been used to induce alignment of corneal keratinocytes (Vrana et al., 2007) and endothelial cells (Zorlutuna et al., 2009). Furthermore, micro-grooved substrates have been used to align nerve cells (Béduer et al., 2012) or within a co-culture system where laminin micropatterns have been used to guide Schwann cells, leading to the formation of aligned neurites (Thompson and Buettner, 2006)*.* Patterns can also be created by using several ECM proteins. A multi-material pattern composed of fibronectin, hyaluronic acid, and collagen has been used to co-culture hepatocytes, fibroblasts, and embryonic stem cells. By exploiting the specific affinity of cells with different patterns, a cellular organization resembling the liver architecture was achieved (Takahashi et al., 2009). Other important applications of patterning rely on the control over cell migration and differentiation. Iannone et al. (2015) demonstrated that by exploiting material surface nanopatterning, it is possible to control the initial spatial positioning and growth of focal adhesions of hMSCs. During the culture on polydimethylsiloxane (PDMS) substrates with arrays of parallel channels having 700 nm width and 1.4 μm pitch, hMSCs underwent a self-organizing differentiation process. When compared with flat surface, after 15 days of culture, hMSCs displayed an overexpression of tenogenic differentiation genes [thrombospondin 4 (THBS4), tenomodulin (TNMD), SMAD8, and scleraxis] and tendon matrix genes (tenascin C, decorin, collagen 1, and collagen 3). This led to the development of 3D tissues with cellular and ECM organization closely resembling that of an embryonic human tendon.

Pioneering studies on cell–materials interaction performed by Harris et al. (1980) established that mechanical stiffness of substrates represents an additional cue that intervenes in controlling the dynamic reciprocity between adhesion plaque growth and substrate deformation. Numerous experimental evidence have corroborated the existence of a regulatory mechanism between local mechanical properties of the materials and cell deformation that represents the foundation of mechano-sensing (Martino et al., 2018). The patterning of mechanical properties has been used to direct cell adhesion and migration in collagen-coated polyacrylamide gels. In particular, at higher gel stiffness, more stable and mature focal adhesions have been observed (Pelham and Wang, 1997). Material stiffness is also involved in controlling cell migration and differentiation. Interestingly, gradients of mechanical stiffness trigger cell migration from soft to rigid regions (Doyle and Yamada, 2016). Many other evidence have reported the possibility to direct stem cell differentiation by modulating material stiffness (Wu et al., 2020; Muncie et al., 2020). Spatial and temporal patterning of mechanical properties may result in crucial relevance in recapitulating morphogenetic processes. Indeed, organs and tissues never develop in isolation but in concert with the surrounding tissues and organs. This implies that each organ is mechanically confined and can impinge upon or pull on other organs. Finally, at the organ and tissue level, regional differences in the mechanical properties arise that serve to pattern cell behavior and differentiation that ultimately drive morphogenetic processes. Such mechanical conditioning is often absent in 3D *in vitro* cultures (Gjorevski et al., 2014). Ultraviolet (UV) ray-sensitive hydrogels can be used as smart material to recreate such mechanical modularity occurring during the morphogenetic process. UV light can modulate, with a micrometric resolution, the mechanical stiffness of matrix regions close to the organoids in order to mimic the expansive growth of the surrounding tissues/organs and consequently induce a controlled local confinement of the growing organoid (Gjorevski et al., 2014). Finally, the ability to produce biomaterials encoding such molecular cues (ECM proteins, GFs, and ECM remodeling enzymes) by arranging them along gradients and patterns with controlled temporal presentation of such signals has increased the possibility to mimic the native context not only in terms of composition but also in terms of cell–ECM reciprocity.

### 3.3 Mimicking native ECMs

**FIGURE 2:**
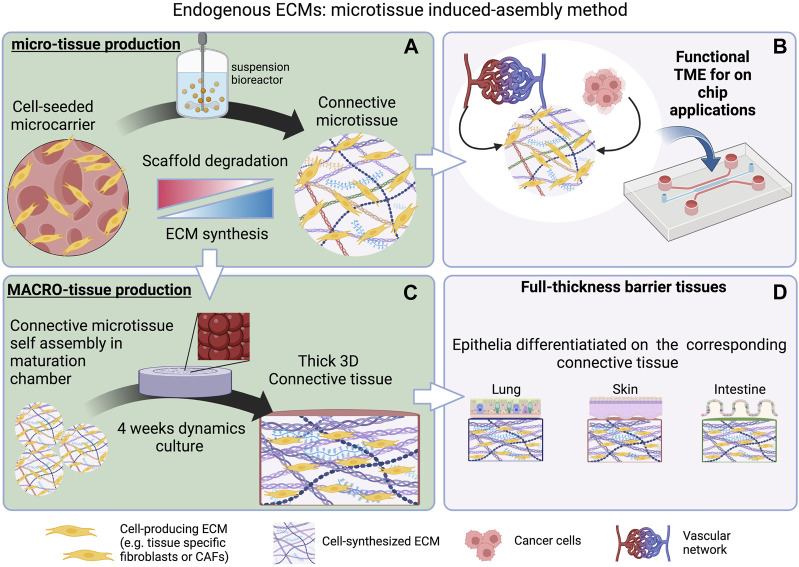
Microtissue-induced assembly method. **(A)** Fibroblasts from different body locations are seeded onto porous MMP-sensitive microcarriers in suspension cultures. Process variables are designed in order to promote the production of ECMs in the bulk porosity and on the surface of themicrospheres. At the end of the process, the microspheres are almost degraded and a connective microtissue that is formed by an endogenous ECM is obtained (Imparato et al., 2013; Urciuolo et al., 2016). **(B)** Such microtissues can represent the connective part of more complex tissues. It can be enriched with vascular endothelial cells forming a functional tumor microenvironment (TME) and cancer cells and can be inserted in microfluidic device (Mazio et al., 2018; Gioiella et al., 2016) for cancer-on-chip applications. **(C)** Connective microtissues can be assembled in thicker connective tissues of >1 mm thick, forming the connective part of **(D)** full-thickness barrier tissues that can be obtained by seeding tissue-specific epithelial cells on top (De Gregorio et al., 2020; De Gregorio et al., 2017; Casale et al., 2016). Created with BioRender.com.

Regardless of the origin of biomaterials (natural or synthetic), their bioactivation degree and spatial arrangement as ECM surrogates still represent an “exogenous” environment, and although representing complex “bio-logic” systems, they are still far from the native context in terms of composition, architecture, functions, signal sequestration, and spatiotemporal presentation. The ECM is a biomaterial designed by nature that underwent over 600 million years of material optimization (Hynes, 2012). Regarding its composition, the ECM involves approximatively 300 bio-macromolecules opportunely arranged depending on the body location or on the tissue development stage. Moreover, the ECM behaves as a “living entity” by undergoing tremendous modifications in concert with the actual needs of the cells, supporting their functions in a specific manner at each stage of tissue development and status (aging, homeostasis, and pathologies). These adaptive/responsive properties occur via modification of spatial organization, stiffening/softening of matrix fibers, compositional modifications, changes in the affinity with water and soluble factors, and changes in the crosstalk between adjacent tissues (e.g., stroma/epithelium interactions) (Kaukonen et al., 2017). Therefore, it is difficult to expect that a biomaterial, even though complex, may be able to capture all the aforementioned features of the cells’ own extracellular space. To overcome such issues, in parallel to the modification of biomaterials, other approaches have been developed in order to tailor the properties of the extracellular space on cell functions. Such approaches can be divided into (i) decellularized ECM-based approach and (ii) endogenous ECM-based approach (Figure 1C; Figure 2). The former is based on the decellularization of the ECM from either native organs or engineered tissues which are then re-seeded with cells (Assunção et al., 2020; Rajab et al., 2020; Solarte David et al., 2022). The latter relies on tissue engineering processes, where somatic cells are induced to synthesize and assemble their own ECM during the *in vitro* culture (self-assembly of cell-synthesized ECM sheets and induced assembly of connective microtissues) (Imparato et al., 2013; Urciuolo et al., 2016). Unlike decellularized ECMs from engineered tissues, in both cell sheet engineering and connective microtissue approaches, the neo-synthesized ECMs are not decellularized but used as living 3D stromal tissue. Both decellularized ECM-based and endogenous ECM-based approaches can be coupled with 3D printing techniques to replicate complex biological architectures.

### 3.4 Decellularized ECMs

Tissue-derived ECMs (tdECMs) are isolated from native organs after the removal of resident cell populations by means of physical, chemical, or enzymatic methods followed by a fixation step with the final aim to preserve the original ECM architecture and composition and to prevent host reactions after implantations (Figure 1C) (Swinehart and Badylak, 2016). The first attempt at preservation of ECM materials with the removal of other mesenteric tissues was reported by Badylak et al. (1995), followed by the production of other tdECMs such as the skin, vascular tissue, heart valves, and bladder (Schmidt and Baier, 2000; Elkins et al., 2001; Chen et al., 2004; Schultheiss et al., 2005). The advent of the perfusion methods for decellularization opened the way (Ott et al., 2010) for the decellularization of whole organs such as the human kidneys, human lungs, heart, and liver (NicholsNiles et al., 2013; Orlando et al., 2013; Mazza et al., 2015). tdECMs possess different advantages when compared with scaffolds obtained with other methods such as (i) preservation of the original organ-specific internal architecture at very high resolution and (ii) partial preservation of native moieties for cell signaling. Despite such advantages, different limitations can be observed. First, the decellularization protocols are not yet standardized as they depend on the organs, and for each organ, the protocol may depend on the size and status. Then, after transplantation, the immunological response still represents an issue. For *in vitro* applications, both recellularization and *in vitro* culture steps are still challenging, and specific bioreactors for cell seeding and tissue cultivation have to be designed. In addition, tdECMs do not allow the full preservation of both tissue and stem cell niches. Indeed, during the decellularization process and the following fixation steps, severe modification of the ECM macromolecules can occur. tdECMs are also produced in the form of bio-inks (Fahimipour et al., 2017; Choudhury et al., 2018; Wang et al., 2021). The possibility of using additive manufacturing or light polymerization methods to arrange cells along complex 3D architectures has the potential to overcome some limitations of the abovementioned approaches such as the difficulty of reseeding the cells in the tdECM. tdECM bioinks (Figure 1 C3) have been developed for different applications such as the heart (Pati et al., 2014), liver (Skardal et al., 2015), skin (Ahn et al., 2017), vascular tissue (Gao et al., 2017), and skeletal tissue (Choudhury et al., 2018; Choi et al., 2016).

Cell-derived ECMs (cdECMs), also called re-engineered ECMs, are obtained *in vitro* by inducing somatic cells to synthesize ECMs with specific properties. Culture conditions can be adjusted to promote ECM deposition by exploiting macromolecular crowding (Zeiger et al., 2012), by inducing hypoxia into cell culture or adding ascorbate in culture media (Hinek et al., 2014). Macromolecular crowding in extracellular culture media, for instance, can be obtained by adding a small amount (1% in volume) of Ficoll^®^ in the culture media. This amount sufficiently crowds the functional proteins of interest to speed up biochemical reactions and assembly (which include enzymatic and polymerization reaction rates, binding and folding kinetics, and gelation and protein fibril formation). Zeiger et al. (2012) demonstrated that macromolecular crowding induces alignment of ECM fibers, cytoskeleton reorganization and alignment, and an increase in deposition of collagen type I in human bone marrow–derived mesenchymal stromal or stem cells. cdECMs are fabricated in different shapes from 2D (standard plates and patterned plates) to 3D (cell sheets layering and pellet aggregations) approaches (Figure 1 C5). The assembled cdECM is then gently decellularized and used in its original format or processed. The resulting cdECMs are used as a coating for both culture dishes and biomaterial surfaces. Integration of cdECM with other materials such as hydrogels provides the opportunity to combine the bioactivity of the ECM with desired geometries and mechanical properties. cdECMs hold the potential to recreate *in vitro* extracellular architectures that are close to the native organs, and for each organ, it is possible to replicate the specific physiological and pathophysiological features. cdECM can be engineered to recreate stem cell and tissue niches. Genetically modified cells have been used to produce recombinant human laminin sheets that, in turn, have been used to trigger the differentiation of either embryonic stem cells (ESCs) or induced pluripotent stem cells (iPSCs) toward pancreatic lineage (Higuchi et al., 2010). Other studies have demonstrated that MSCs reseeded on MSC-derived ECMs were fprotected from oxidative stress and senescence, with increased proliferation and stemness preservation. In this direction, stem cell–derived ECMs have been used to maintain the phenotype of progenitor neural cells, embryonic stem cells, and hematopoietic stem cells. In a similar fashion, tissue niches can be replicated in order to culture tissue-specific cells such as chondrocytes (Yang et al., 2018), (Zhang et al., 2021b), podocytes (Satyam et al., 2020), and Schwann cells (Haring et al., 2019). Often, aligned extracellular structures are required if one wishes to recapitulate specific cell–ECM interactions occurring during morphogenesis or pathological conditions. For example, during epithelial branching morphogenesis, local anisotropy of collagen affects the orientation of epithelial branching (Brownfield et al., 2013). In tumors, aligned collagen fibers activate fibroblasts which strengthen the alignment of the ECM fibrous structure by increasing contractile forces. Such structural remodeling facilitates cancer cell invasion and consequently promotes intravasation (Brownfield et al., 2013; Han et al., 2016; Conklin et al., 2018; Emon et al., 2018; Zanotelli et al., 2018). To accomplish such needs, nanopatterned surfaces are used to induce the alignment of somatic cells producing highly oriented and packed collagen structures. After decellularization, reseeded fibroblasts show cytoskeleton organization, oriented growth of protrusions, and focal adhesions along with the aligned matrix (Taufalele et al., 2019). Finally, culturing cancer cells in cdECMs obtained by using stromal cells from invasive tumors has shown to recapitulate increased malignancy and drug resistance in comparison with cancer cells cultured on other non-tumor cdECMs (Satyam et al., 2020; Hoshiba and Tanaka, 2013; Hoshiba, 2018). Overall, when compared with functionalized scaffolds, decellularized ECMs (both tdECMs and cdECMs) are conceived to retain all the information as much as possible that the native ECMs present to cells *in vivo.* This is also demonstrated by transcriptome analyses as different works report that the matrisome (the ECM signatures) related to the decellularized ECMs is quite similar to the native counterpart and the presence of important key regulators of cell functions can be retained such as collagen, glycoproteins, proteoglycans, ECM-affiliated molecules, and secreted factors (Yuan et al., 2018; Ragelle et al., 2017). Yuan et al. (2018) demonstrated that after decellularization of the nucleus pulpous niche obtained *in vitro*, GAGs and collagen type II, and the cytoskeletal protein keratin 19, were retained. In addition, TGF-β and its membrane-bound receptor TGF-β receptor I were partially retained. Finally, other ECM proteins were retained after decellularization such as prolargin (PRELP), lactadherin (MFGE8), biglycan (BGN), fibromodulin (FMOD), hyaluronan, and proteoglycan link protein 1 (HAPLN1). Interestingly, after seeding such decellularized ECM with dermal fibroblasts, it was observed that the specific microenvironment could control the fate of dermal fibroblasts. The latter primarily produce collagen type I but not collagen type II. However, when these cells were cultured in such decellularized ECM, the expression of collagen type I decreased and the expression of collagen type II increased according to the *in vivo* situation. Furthermore, by using different cell types, the cdECM possesses a different matrisome (Ragelle et al., 2017), highlighting the possibility to preserve tissue-specific cell–ECM interactions. Although decellularized ECMs allow recreation of specific tissue architecture and compositions, with the possibility to rebuild complex 3D structures by using bioprinting, several limitations still affect the cdECMs. Indeed, they are often in the form of sheets having a thickness of approximately 20 μm that are difficult to handle and are mainly used as functional coatings. In many applications, cdECM bioinks have to be coupled with synthetic materials to improve mechanical properties. In addition, decellularization agents may compromise many ECM macromolecules. Indeed, Triton X and sodium dodecyl sulfate (SDS) have been shown to remove GAGs, GFs, and collagen. Prolonged exposure to trypsin can damage the ECM ultrastructure and deplete collagen, laminin, elastin, fibronectin, and GAGs.

### 3.5 Endogenous ECMs

In different applications, the cell-derived ECMs are used as living connective tissue composed of cells and their own ECMs without the decellularization step. The living tissues that are realized in this way are then “enriched” with other cell types (cancer cells, epithelial cells, endothelial cells, *etc.*) with the final aim to obtain more complex biological entities that in their final configuration are characterized by a connective compartment composed of fibroblasts embedded in their own ECM. These *in vitro*–formed ECMs can be defined as endogenous ECMs since the cells and their own ECM are not decoupled and evolve together during the entire *in vitro* tissue genesis process. The tissue engineering strategies that fall in this category comprise self-assembly of cell sheets (Roy et al., 2020) and induced assembly of connective microtissues (Brancato et al., 2017a).

The *cell sheets* are obtained by culturing stromal cells (fibroblasts or MSCs) in 2D culture plates for up to 28 days of culture. Stromal cell sheets can be stacked to obtain thicker tissues and are further covered by epithelial cells to form full-thickness barrier tissues. In parallel, stromal cell sheets can be populated by organoids, cancer cell spheroids, and either vascular or lymphatic endothelial cells (Roy et al., 2020). An example of engineered tissues realized by using this technique is human skin, melanoma, bladder cancer, and uveal melanoma (Roy et al., 2020). By comparing this approach with others based on biomaterial functionalization or decellularization on pre-existing ECMs, it must be recognized that the assembly of cell sheets brings different advantages. They do not require any exogenous materials and the arrays of signals useful for the maintenance of tissue functions are secreted by the cells themselves. In addition, if compared with both organ-derived ECMs and cdECMs discussed above, the absence of decellularization steps guarantees the full preservation of signals present in the EMCs. An example of the implementation of this approach is basal cell carcinoma (BBC) constructs obtained by Roy et al. (2020) by seeding malignant keratinocytes on an endogenous sheet of the dermis that showed morphological features close to the native BCC cancers: nests of basaloid cells surrounded by a fibromyxoid stroma. Tumoral keratinocytes also displayed abnormal proliferating phenotypes in terms of divergent expression patterns of K10 and K15. Furthermore, the use of fibroblasts or MSCs harvested by tissue biopsies allows the recreation of site- and patient-specific ECMs capable of mimicking both physiologic and pathologic conditions. A limitation of the cell sheet approach is their dimension (approximately 20 μm) and the necessity to layer different cell sheets to obtain a thicker connective tissue. Finally, the cell sheets are highly cellularized, despite the moderate cell density featuring the connective part of some organs such as the skin and other barrier tissues. This implies a high metabolic request which limits the number of sheets that can be assembled without necrosis risk. Together with high cell density, the ECM proteins are much highly packed and denser when compared with their native counterparts. This is probably due to the high cell density featuring the 2D cultures, resulting in an increased traction force on ECM proteins leading to their compaction and densification.

**FIGURE 3:**
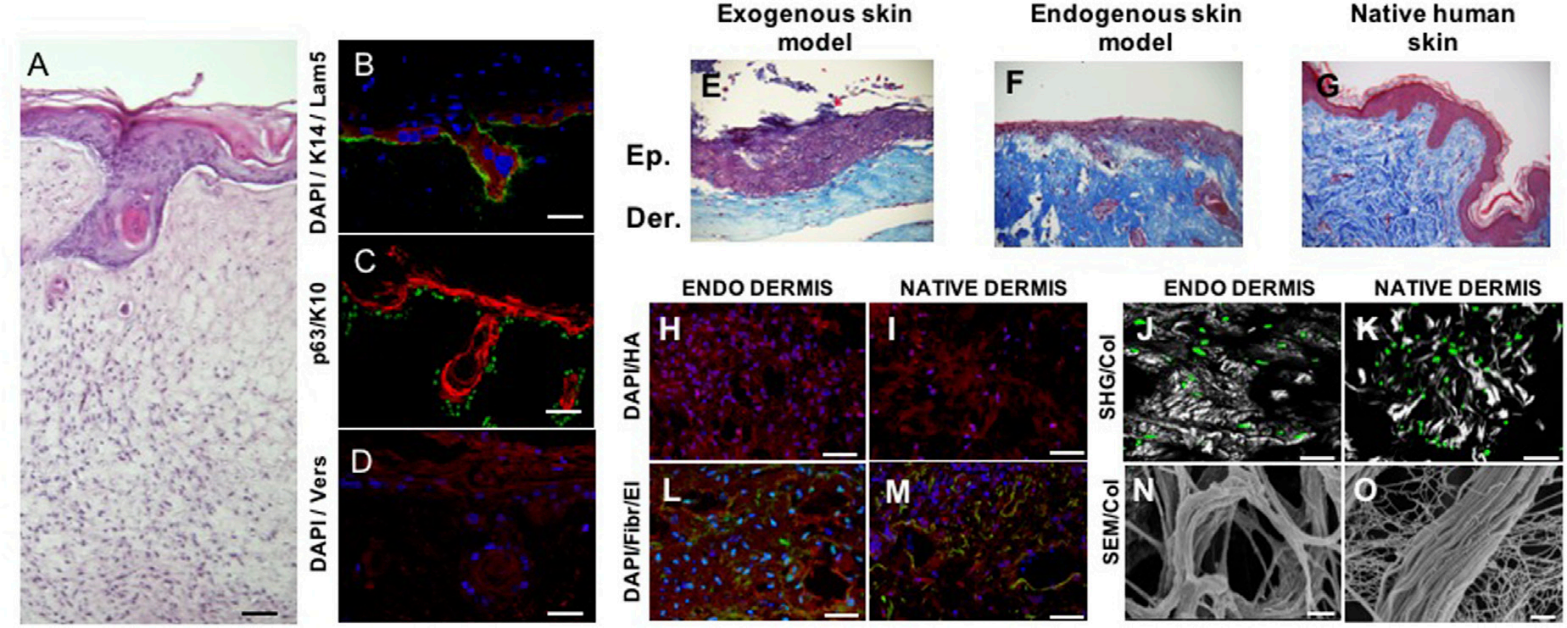
Spontaneous epithelial mesenchymal crosstalk *in vitro*. Full-thickness human skin equivalent presenting spontaneous formation of follicle-like structures **(A)**. Dermis compartment is made of completely endogenous human dermis obtained by induced-assembling of connective microtissue; epidermis is obtained by culturing primary human keratinocytes on the top of dermis (Kanchanawong and Calderwood, 2023). Dermis–epidermis interface—presenting K14 in red, laminin in green, and DAPI in blue **(B)**; p63 in green and K10 in red **(C)**; versican in red and DAPI in blue **(D)**. Dermis–epidermis interface in exogenous collagen matrices (exogenous skin model, **(E)**, endogenous 3D matrices (endogenous skin model, **(F)**, native human skin **(G)**. Comparison of the composition and architecture of endogenous 3D dermis (endodermis) vs. native human dermis: cell-synthesized hyaluronic acid in red **(H, I)**; cell-synthesized fibronectin (red) and elastin (green) **(L, M)**; cell-synthesized collagen in gray **(N, O)**; cells in green **(J, K)**; and SEM **(N, O)**. SHG = second harmonic generated signal from multiphoton microscopy. Scale bar: 50 μm in **(A)**; 40 μm in **(B)**; 50 μm in **(C, D, H, I, L, M, J, K)**; 2 μm in **(N, O)**. Image adapted from reference Casale et al. (2018) with permission.

The *microtissue-induced assembly method* (Figure 2) is based on a similar concept of the cell sheet self-assembly but the ECM is formed in a spherical geometry instead of a planar one. Fibroblasts derived from different body districts are seeded onto porous MMPs-sensitive gelatin microbeads in a suspension bioreactor (i.e., spinner flasks) (Imparato et al., 2013). Once adhered on to the microbeads' surface and in the inner porosities, both hydrodynamic and biochemical culture conditions are adjusted in order to induce fibroblasts to produce their own ECM which will be present in the bulk of the microbeads within the pores and as a rim surrounding the surface of the microbeads. Such an entity is known as microtissue precursor (μTP) and depending on the fibroblasts’ origin, they can be divided into dermis-μTP (Urciuolo et al., 2016), intestine-μTP (De Gregorio et al., 2020), tumor-μTP (Brancato et al., 2017a), cardiac-μTP (Totaro et al., 2016), and cervix-μTP (De Gregorio et al., 2017). The μTPs are approximatively 500 μm in diameter and can be used in different ways. In tissue-on-chip applications, with the aim to provide a functional tumor microenvironment, the μTPs can act as tissue-specific connective tissue (Figure 2B). Once enriched with endothelial cells, the μTPs form a complex stromal compartment in which cancer cell invasion, ECM remodeling, and vascular network re-organization can be replicated (Mazio et al., 2018). Used as building blocks to obtain thick site-specific connective tissues, the μTPs can assemble via cell–cell and ECM–ECM interactions between the rim of cell-synthesized ECM surrounding adjacent μTPs. In this way, it is possible to fabricate large and thick (>1 mm) site-specific connective tissues used to build full-thickness barrier organs (Figures 2C, D) (De Gregorio et al., 2020; De Gregorio et al., 2017; Casale et al., 2016; Casale et al., 2018). When compared with the self-assembly of cell sheets, different advantages characterize the microtissue-induced assembly. From an operational point of view, it is difficult to carry out overlapping of different sheets since each sheet has to be detached from the culture plate and then overlapped. Often, this operation leads to mechanical damage of the sheets. On the contrary, connective microtissue can be suspended in a syringe and cast in a maturation space in which their bio-sintering takes place. Once packed in the maturation space, microtissue assembly allows the formation of a thicker dermis when compared with that of cell sheet—1 mm (Casale et al., 2018) vs. 100 μm (Roy et al., 2020). Moreover, the 3D growth featuring the assembly of microtissues allows the recreation of more physiological spatial arrangements possessing (i) a lower cell-to-ECM ratio if compared with 2D cell sheets and (ii) architectural features of the collagen network closer to their native counterparts (Figure 3) (Casale et al., 2016; Casale et al., 2018). Specific applications such as the microtissue assembly method to replicate *in vivo–*like morphogenetic processes and ECM modification during pathological events will be discussed in Section 4.

## 4 *In vivo*–like morphogenetic process and ECM dynamics in engineered tissues

Endogenous ECM-based engineered tissues possess capabilities to mimic the ECM dynamics of native tissues, and therefore allow the replication of some biological processes *in vitro* that are not observable in traditional exogenous scaffold-based models. In the following section, some examples are discussed, highlighting the role of cell–ECM crosstalk in guiding morphogenesis and progression of pathologies.

### 4.1 Epithelial–mesenchymal interaction in hair regeneration

We have already addressed the pivotal role of the ECM in the development of branched organs (Fata et al., 2004) Indeed, the spatiotemporal regulation of ECM provides cells with permissive and instructive morphogenic signals, triggering and guiding the development of branched structures. Therefore, *in vitro* models must recreate 3D extra cellular context as similar as possible to the native one, to drive cells toward the formation of ordered structures that faithfully recapitulate those found in the human body (Casale et al., 2016; Zhang and Khademhosseini, 2015). The human hair follicle is an example of these ordered structures; it is a complex skin appendage that in adults appears as a kind of “epidermis branch” growth downward in the dermis. Its morphogenesis occurs during embryogenesis and equally depends on different stem cells and well-orchestrated epithelial–mesenchymal interactions. Cell–ECM interactions in the human hair follicle are crucial to maintain the specific cell phenotype and their role within the individual compartments, and they are also involved in physiological and pathophysiological alterations (Watt, 2016; Abreu and Marques, 2022). The hair follicle morphogenesis starts during embryogenesis when a dermal signal derived from mesodermal cells induces epidermal progenitors to create the placode. In response, an epithelial signal stimulates the dermal cells to cluster below the placode, forming the dermal condensate. A further dermal signal triggers the placode proliferation and invasion in the dermis, until the formation of the hair peg. Following a continuous downward proliferation, the epithelial cells envelop the dermal condensate, which evolves into the dermal papilla. The epithelial–mesenchymal interactions established between the hair peg and dermal papilla further promotes the proliferation and differentiation of the epithelial cells into the different structural layers of the mature hair follicle, until the formation of the hair fiber (Korosec and Lichtenberger, 2017). Unlike what happens in some other mammals, in humans, no hair follicles are naturally formed after birth, therefore when a hair disease occurs or in the case of full-thickness skin defects, the hair follicle cannot regenerate on their own. In this scenario, skin substitutes can be considered the ideal treatment, but existing engineered skin models present only the epidermal and dermal layers and have limited regenerative capacity, preventing appendage reformation (Abreu and Marques, 2022). The more relevant aspects associated with the creation of hair follicle regenerative microenvironments *in vitro* involve the typology of cell source used (the use of relevant mesenchymal and epithelial cells and the ability to maintain their key properties) and the importance to replicate an adequate supportive ECM. The relevance of the former has been widely demonstrated in several articles (Abaci et al., 2018) and, in particular, by the results obtained by Lee et al. (2020) who succeeded in producing hair-bearing skin organoids cultured and matured over 140 days *in vitro* by aggregating human embryonic or iPSC in Matrigel enriched with a complex mix of GFs. This is the most comprehensive *in vitro* imitation of human hair follicle accomplished so far and emphasizes the significance of combining inductive dermal papilla cells and immature epithelial cells with a high proliferative ability for the imitation of hair follicle cellular compartments, driving efforts toward the preservation of the trichogenic capability of human cells *in vitro* (Abreu and Marques, 2022). Due to the scope of this review, we will go more in detail on the other crucial aspects which regard the relevance of replicating a supportive ECM for hair follicle morphogenesis. The ECM has a key role in directing hair growth and maintaining cell function and the fact that follicles are usually not found in the skin that contains scar tissue represents a further proof of the repository and regulatory role of the ECM in providing the cells with the correct instructive signals to trigger the folliculogenesis process (Casale et al., 2018; Gharzi et al., 2003). In spite of the research conducted in this field, its practical application is still limited, and existing bioengineered hair follicle models remain rudimentary and inadequate in terms of accurately replicating the intricate environment of the ECM or the signaling molecules responsible for hair growth (Abreu and Marques, 2022; Casale et al., 2018). In addition, the literature regarding hair regeneration continues to be dominated by murine cell studies or chimeric human–murine combinations, instead of purely human techniques. As a result, human hair regeneration that is promoted entirely from human adult cells is yet to be achieved (Abreu and Marques, 2022). On this line, our group succeeded in producing human skin equivalent from adult cells in which follicle-like structures were spontaneously generated (Figure 3 A) without adding MSCs. The human skin equivalent was featured by a dermal compartment built-up through connective microtissue assembly in which human dermal fibroblasts were guided to produce and assemble their own ECM presenting several of the complex macromolecules that characterize the native ECM (Lombardi et al., 2017; Imparato et al., 2017; Urciuolo et al., 2016; Casale et al., 2016; Casale et al., 2018) (Figures 3H–O; Casale et al., 2018). More in detail, the connective microtissues were fabricated by using human primary fibroblasts seeded onto porous gelatin microcarriers in suspension bioreactors. During 1 week of culture in suspension, the fibroblasts adhered onto microcarriers and started to produce their own ECM as described in the Section “*microtissue-induced assembly method*”. Furthermore, the microtissues were placed in the cylindrical mold and kept under dynamic culture conditions to allow their “bio-sintering” through cell–cell and ECM-ECM contact. During the bio-sintering process, the porous microcarriers underwent degradation due to MMPs activity, and after 4 weeks, the microtissue formed a dense and compact dermis-like tissue composed of fibroblasts embedded in their own assembled ECM (Urciuolo et al., 2016; Casale et al., 2018; Urciuolo et al., 2016). At last, the epidermis was produced by traditional air/liquid interface culture by seeding adult keratinocytes on the dermis, and at 2 weeks of culture, the epidermal cells started to grow downward in the dermis resembling the first step of hair follicle embryogenesis. The formation of such folliculoid structures by using dermal and epidermal human adult cells was very surprising, and we hypothesized that our skin models provided a physiological environment that could preserve the stemness of the germinal cell layers and address the fate of adult cells toward the genesis of appendage-like structures. We deeply investigate the morphology and composition of our human skin model by performing histological, immunofluorescence, and immunohistochemical analyses (to detect specific dermis and epidermis markers). To figure out the role of the endogenous dermis on hair follicle morphogenesis, a comparison with standard skin models obtained by fibroblast-populated animal collagen as the dermal compartment was made (Figures 3E, F). Even if the same human adult dermal and epidermal cells were used to produce the two skin models, they resulted in two constructs featured by dermal compartments that were deeply different. In our model, the dermal ECM was of endogenous nature, which was synthesized and assembled by the human fibroblasts, whereas in the case of the standard model, it was an animal collagen network that underwent contraction but was not physiologically responsive to cell remodeling (Casale et al., 2018). Our results have demonstrated that the nature of the dermal environment (Figures 3H–O) strongly affects the behavior of the epithelial cells and in turn the morphogenesis of the epidermis, highlighting that only in our model, the presence of follicle-like structures and the convolute profile of the dermal–epidermal junction could be observed (Figures 3E, F). By analyzing the molecular composition and organization of the ECM in the two models, the detection in our skin model of the versican in the dermal ECM, epidermal compartment, and follicle-like structures and the lack of such molecules in the exogenous collagen-based model appeared particularly interesting. Indeed, versican is a proteoglycan expressed *in vivo* eccentrically toward the hair follicle in the anagen phase, in the proliferating zone of the epidermis, and in association with the elastic network of the dermis (Casale et al., 2018; Zimmermann et al., 1994; Kishimoto et al., 1999). Its presence in our model suggested that fibroblasts and keratinocytes could provide a highly hydrated matrix facilitating inward movements of proliferating keratinocytes into appendage-like structures. On the contrary, the absence of versican in the exogenous collagen-based model demonstrated the inability of the dermal cells to assemble this inductive signal in a not physiologically relevant *in vitro* model. Such results support the hypothesis that the endogenous dermal ECM of our model could replicate the repository and regulatory role of the native counterpart in guiding tissue morphogenesis and promoting the physiological dermal–epidermal interaction, resulting in a skin model with unexpected features. In addition, it is relevant to highlight that in other endogenous ECM-based approaches (Roy et al., 2020), the formation of such structures has never been reported. This is probably due to the highly packed nature of the ECM in the cell sheets which results in a dysfunctional architecture of the ECM macromolecules hindering a physiological dermis/epidermis crosstalk. Taken together, these results shed light on the fundamental role of the 3D environment context, raising some doubts on the use of both exogenous and cell-synthesized matrices that lack a well-organized ECM structure for building-up functional organotypic models *in vitro*.

### 4.2 *In vitro* replication of ECM modifications during pathological events

*Replication of TME dynamics.* Relevant biological phenomena related to ECM dynamics are, but not restricted to, alterations of the tumor microenvironment (TME) during cancer progression, modifications of the ECM due to external stimuli (e.g., UV exposure), intrinsic stimuli (e.g., aging), wound healing, or inflammations. With the term modification and/or alteration, we refer to the variations in compositions, architecture, biophysical properties, and spatial re-organization of signals occurring at the ECM level. To the best of our knowledge, by using exogenous matrices, the recreation of specific dynamics involving the TME has been only partially recapitulated. Often, the most relevant phenomenon that exogenous matrices have displayed is related to the stiffening and alignment of preexisting exogenous fibers due to traction force exerted by fibroblasts or cancer-activated fibroblasts (CAFs) (Frantz et al., 2010; Liu et al., 2019; Gupta et al., 2020; Shinsato et al., 2020). By contrast, endogenous-based ECMs (self-assembly of cell sheets—Roy et al., 2020 or connective microtissue approach—Brancato et al., 2017b) have proved to display ECM dynamics closer to the *in vivo* situation. Brancato et al. (2017b) have demonstrated that when connective microtissues were obtained by inducing fibroblasts or CAFs to produce their own extracellular space, the final microtissues displayed different ECMs in terms of composition and architecture. In particular, the ECM from CAFs was richer in collagen, hyaluronic acid, and elastin than the ECM obtained from normal fibroblasts, resembling the composition of the activated ECMs during tumor progression. Moreover, transport and mechanical properties and metabolic activities were different. CAF-derived ECMs are stiffer and the final microtissues display an increased cell-specific oxygen consumption rate. This demonstrates the possibility to recreate a site- and status-specific TME. By following this approach, several kinds of cancer models such as pancreatic ductal adenocarcinoma (PDAC) (Brancato et al., 2017a), vascularized breast cancer (Mazio et al., 2018), and breast cancer-on-chip (Gioiella et al., 2016) have been replicated, each one featuring a stromal compartment built up by site-specific normal fibroblasts or CAFs. The endogenous-based 3D PDAC model could display a desmoplastic reaction at the molecular, cellular, and most importantly, extracellular levels (Brancato et al., 2017a). The desmoplastic reaction is driven by quiescent pancreatic stellate cells, which are activated by cancer cells to acquire a CAF phenotype, expressing some markers as smooth muscle α-actin (α-SMA) and platelet-derived GF β (PDGFβ) (Malik et al., 2015). In the work of Brancato et al. (2017a), connective microtissues that were obtained by using normal fibroblasts were coupled with PT45 (a human PDAC cell line carrying mutated KRAS, TP53, and CDKN2) to obtain a 3D PDAC model composed of malignant cells invading a living connective tissue. When compared with the control group (connective microtissues without PT45) in the 3D PDAC model, it was observed that an overexpression of α-SMA and PDGFβ indicated a phenotypic change in fibroblasts. At the extracellular level, important *in vivo*–like modifications were detected in the 3D PDAC models in terms of overexpression of collagen and GAG content and modification of collagen network architecture. In addition, molecular investigation performed by qRT-PCR and immunophenotypic staining demonstrated the upregulation of ECM-related genes such as collagen III, collagen IV, collagen V, MMP-2, and periostin in the 3D PDAC model. In other studies, to mimic the breast cancer invasion *in vitro*, connective microtissues were made starting from by breast fibroblasts, enriched with endothelial cells and MCF-7 malignant cell lines (Mazio et al., 2018). It was shown that the presence of endogenous ECMs mediated the formation of intratumor heterogeneity in terms of ECM rearrangement and vascular network remodeling. In fact, in the same microtissue, both randomly distributed and highly oriented collagen fibers could be recognized, and each collagen architecture was related to specific cancer cell configuration (e.g., single file and solid strand) (Mazio et al., 2018). In a different application, a model of breast cancer invasion on-chip was replicated (Gioiella et al., 2016). Malignant breast epithelial cells were placed in contact with endogenous connective microtissues, and their invasion was observed in real time by means of multiphoton microscopy in order to evaluate the collagen fiber re-organization. It was observed that collagen network showed *in vivo–*like variations in terms of architecture and composition. Other than activation of normal fibroblasts into CAF lineage, the activation of the ECM components in terms of hyaluronic acid and fibronectin overproduction was observed (Gioiella et al., 2016). Interestingly, relevant architectural and transport properties in the connective compartment were observed. The correlation length of the collagen network, evaluated by analyzing the textural features of the cell-synthesized collagen (MengWang et al., 2023), increased during cancer cell invasion according to the *in vivo* situation (Burke et al., 2012; Burke and Brown, 2014; MengWang et al., 2023). Finally, the transport properties, in terms of diffusivity of large macromolecules in the ECM, changed during cancer cell invasion.

*Replication of dermis damage and repairing process.* The human dermis displays relevant modifications after exposure to UV light and during the repairing process of deep wounds. The inflammation occurring after UV exposure leads to modifications of ECM composition and architecture. Such modifications play a crucial role in skin functions: the departure of ECM composition, architecture, and mechanical properties from the physiological status is responsible for skin aging, which is ultimately related to a wide range of pathological events (Rittié and Fisher, 2015). In this scenario, beyond cellular events such as reactive oxygen species (ROS) release and overexpression of MMPs and TGF-β, the possibility to study the evolution of the extracellular space is advantageous for the development of molecules that aims at restoring the physiological ECM features. In this direction, we demonstrated that a full-thickness human skin equivalent, featuring an endogenous dermis obtained by means of the microtissue assembly method, showed a variation in ECM composition and remodeling after UV exposure in terms of collagen and hyaluronic acid. In addition, collagen displayed a transition from a fine network (in the untreated tissues) to a coarse network (in the UV-exposed tissues), while hyaluronic acid displayed accumulation in the extracellular space after the exposure. In parallel, the full-thickness skin model featuring an exogenous collagen dermis subjected to the same dose of UV did not show any variation at the ECM level (Imparato et al., 2017; Casale et al., 2018).

**FIGURE 4:**
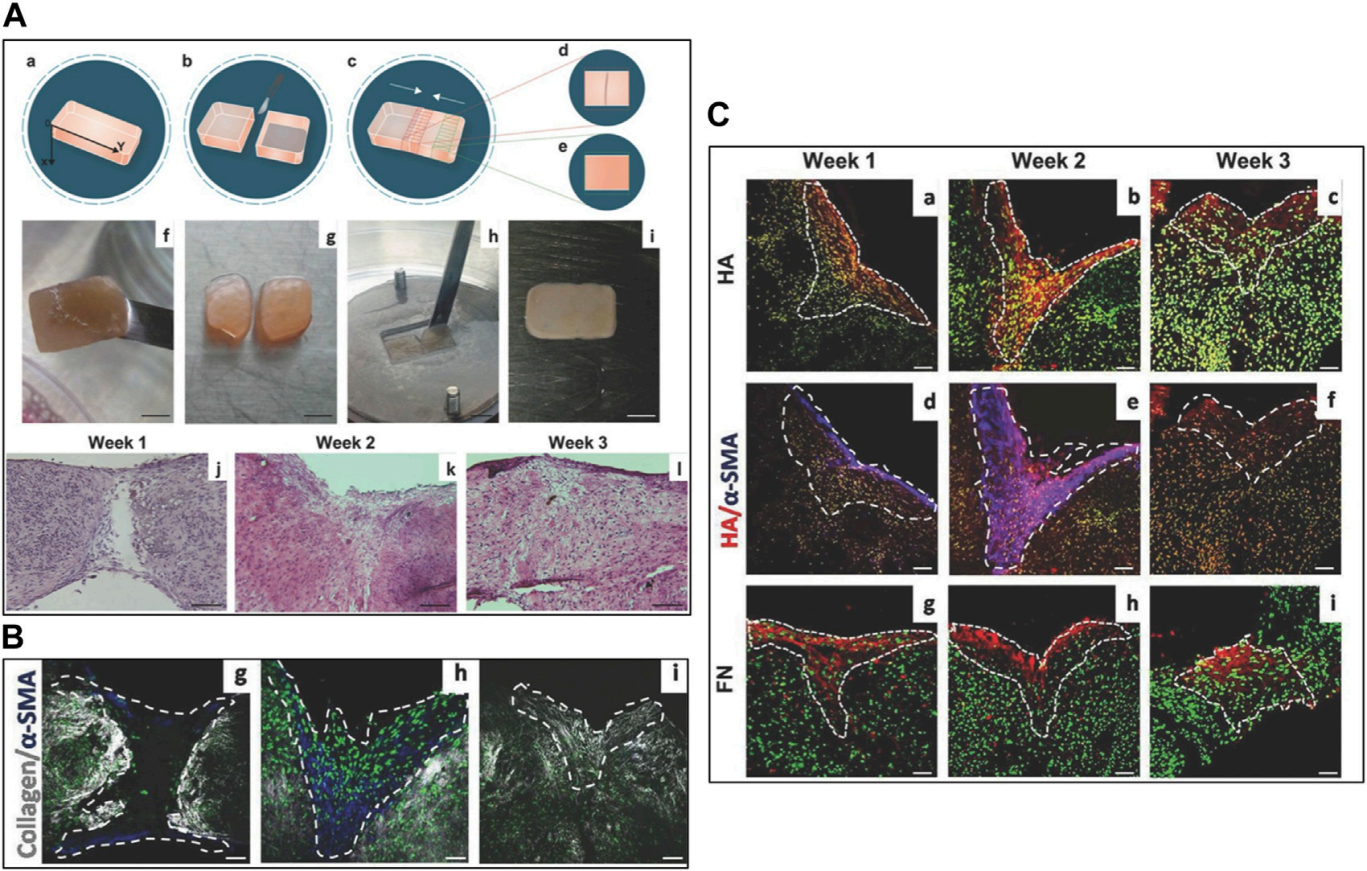
*In vitro* replication of ECM remodeling during wound closure by using engineered endogenous human dermis: **(A)** experimental set-up and tissue morphology over 3 weeks. (a–i) experimental set-up for performing the cut and to mimic primary intention wounds. (j–l) Histology (hematoxylin and eosin) assess the time evolution of the deposition of new ECM over 3 weeks. **(B)** g–i SHG signals from preexisting and neo-formed endogenous collagens network in gray, α-SMA in blue, and cell nuclei in green during the closure process; **(C)** a–c time evolution of neo-formed hyaluronic acid (HA) in red and cell nuclei in green; d–f time evolution on neo-formed HA and α-SMA shown as overlapped signal in purple; g–i time evolution of neo-formed fibronectin (FN) in red. Image adapted from Lombardi et al. (2017) with permission. SHG = second harmonic generated signal from multiphoton microscopy.

The remodeling of the ECM during the healing of deep wounds also plays a crucial role in the formation of scar tissues. The recreation of the extracellular events occurring during the closure of the human dermis should represent the starting point for the development of strategies that aim at avoiding the formation of severe scars. To this aim, the endogenous dermis obtained by microtissue assembly approach has been proved to recapitulate ECM remodeling steps occurring *in vivo* during the wound-healing process (Figure 4). After mechanical damage, the endogenous human dermis (Imparato et al., 2017) shows the activation of fibroblasts toward a myofibroblast phenotype (Lombardi et al., 2017). Then, during the closure period, it is possible to observe the deposition of the neo-synthesized ECM in the damaged zone in terms of key players of the wound-healing process such as collagen, fibronectin, and hyaluronic acid (Figure 4C) (Urciuolo et al., 2022). Fibroblasts migrated from the non-wounded region to the wounded one acquiring a myofibroblastic phenotype. Collagen in the damaged zone was shown to increase over a period of 3 weeks, and new collagen fibrils appeared orthogonally oriented to the direction of the wound edges. Hyaluronic acid showed a transient overexpression while fibronectin increased over time. These results show the possibility to recreate *in vitro* the complexity of the wound-healing process occurring at both the cellular and extracellular levels.

In parallel, fibroblasts-populated exogenous matrices have also been used to mimic the phenomena involved in dermis wound healing. Sakar et al. (2016) provided experimental evidence on mechanistic phenomena occurring between fibroblasts and collagen fibers during the closure of a mechanically induced gap. By observing in real time how fibroblasts remodeled the gap, it was possible to show that the closure was driven by contractility rather than fibroblast proliferation. The contractility, in turn, governed the fibroblast migration along the wound edges and newly formed fibrillar.

Fibronectin was assessed as serving in the provisional matrix for the cells to close the gap, mimicking the early stages of the wound closure. The assay recapitulated very important events occurring at both the cellular and extra-cellular levels. Nevertheless, the relevance of the model is partly limited to the use of cells of animal origin (i.e., NIH 3T3) and by the short experimental time that hinders the possibility to follow the synthesis and assembly of other ECM components such as collagen and hyaluronic acid (Sakar et al., 2016).

## 5 Conclusion

We have reported that cells decoupled by their native context and reseeded in an exogenous scaffold are not able to restore the physiological cell–ECM interaction, which is crucial for replicating all biological processes occurring in the native tissues. In addition, decellularization of cell-derived ECMs should be considered an exogenous approach since the cells are not embedded in their own 3D context and cannot recognize the surrounding environment as native. The use of an endogenous-based approach seems to be more promising if one wishes to fully replicate the functions of native tissues *in vitro*. This has been analyzed in the light of relevant biological phenomena where the ECM matters: *in vitro* replication of spontaneous morphogenetic process as well as time and space evolution of the ECMs after damages. Endogenous-based approaches can potentially lead to the fabrication of living tissues possessing functionalities closer to their native counterparts. This will improve the outcome of treatments such as wound healing and organ regeneration. Moreover, fully endogenous tissues possess a superior biological relevance with tremendous advantage for the organ-on-chip applications. Although the production of endogenous tissue is longer than in other approaches, the preservation of the cell–ECM interaction allows the *in vitro* replication of the ECM-related phenomena that plays a crucial role in pathologic events such as fibrosis, TME transformation, and ECM remodeling. The use of endogenous engineered tissues for *in vitro* screening provides a more physiologically relevant environment by better mimicking the complexity of living systems and leading to more accurate predictions of drug efficacy and toxicity. Additionally, the use of patient-specific engineered tissues has the potential to offer personalized and more effective treatments. Despite some challenges, the benefits of this approach make it a promising area of research and development.

## References

[B1] Abaci H. E. Coffman A. Doucet Y. Chen J. Jacków J. Wang E. (2018). Tissue engineering of human hair follicles using a biomimetic developmental approach. Nat. Commun. 9 (1), 5301–5311. 10.1038/s41467-018-07579-y 30546011 PMC6294003

[B2] Abbasian M. Massoumi B. Mohammad-Rezaei R. Samadian H. Jaymand M. (2019). Scaffolding polymeric biomaterials: Are naturally occurring biological macromolecules more appropriate for tissue engineering? Int. J. Biol. Macromol. 134, 673–694. 10.1016/j.ijbiomac.2019.04.197 31054302

[B3] Abreu C. M. Marques A. P. (2022). Recreation of a hair follicle regenerative microenvironment: Successes and pitfalls. Bioeng. Transl. Med. 7 (1), 102355–e10318. 10.1002/btm2.10235 PMC8780054 35079623

[B4] Ahn G. Min K. H. Kim C. Lee J. S. Kang D. Won J. Y. (2017). Precise stacking of decellularized extracellular matrix based 3D cell-laden constructs by a 3D cell printing system equipped with heating modules. Sci. Rep. 7 (1), 8624–8711. 10.1038/s41598-017-09201-5 28819137 PMC5561246

[B5] Alexander J. Cukierman E. (2016). Stromal dynamic reciprocity in cancer: Intricacies of fibroblastic-ECM interactions. Curr. Opin. Cell. Biol. 42, 80–93. 10.1016/j.ceb.2016.05.002 27214794 PMC5064819

[B6] Salerno A. Netti P. A. (2021). “Review on computer-aided design and manufacturing of drug delivery scaffolds for cell guidance and tissue regeneration,” Front. Bioeng. Biotechnol. 9, 682133–682219. 10.3389/fbioe.2021.682133 34249885 PMC8264554

[B7] Asghari Sana F. Çapkın Yurtsever M. Kaynak Bayrak G. Tunçay E. Ö. Kiremitçi A. S. Gümüşderelioğlu M. (2017). Spreading, proliferation and differentiation of human dental pulp stem cells on chitosan scaffolds immobilized with RGD or fibronectin. Cytotechnology 69 (4), 617–630. 10.1007/s10616-017-0072-9 28653139 PMC5507842

[B8] Hoffman A. S. (2012). “Hydrogels for biomedical applications,” Adv. Drug Deliv. Rev. 64, 18–23. 10.1016/j.addr.2012.09.010 11755703

[B9] Assunção M. Dehghan-Baniani D. Yiu C. H. K. Später T. Beyer S. Blocki A. (2020). “Cell-derived extracellular matrix for tissue engineering and regenerative medicine,” Front. Bioeng. Biotechnol. 8, 602009–602010. 10.3389/fbioe.2020.602009 33344434 PMC7744374

[B10] Badylak S. F. Tullius R. Kokini K. Shelbourne K. D. Klootwyk T. Voytik S. L. (1995). The use of xenogeneic small intestinal submucosa as a biomaterial for Achille’s tendon repair in a dog model. J. Biomed. Mat. Res. 29 (8), 977–985. 10.1002/jbm.820290809 7593041

[B11] Bao M. Xie J. Piruska A. Huck W. T. S. (2017). 3D microniches reveal the importance of cell size and shape. Nat. Commun. 8 (1), 1962–2012. 10.1038/s41467-017-02163-2 29213086 PMC5719012

[B12] Béduer A. Vieu C. Arnauduc F. Sol J. C. Loubinoux I. Vaysse L. (2012). Engineering of adult human neural stem cells differentiation through surface micropatterning. Biomaterials 33 (2), 504–514. 10.1016/j.biomaterials.2011.09.073 22014459

[B13] Booth A. J. Hadley R. Cornett A. M. Dreffs A. A. Matthes S. A. Tsui J. L. (2012). Acellular normal and fibrotic human lung matrices as a culture system for *in vitro* investigation. Am. J. Respir. Crit. Care Med. 186 (9), 866–876. 10.1164/rccm.201204-0754oc 22936357 PMC3530219

[B14] Braghirolli D. I. Steffens D. Pranke P. (2014). Electrospinning for regenerative medicine: A review of the main topics. Drug Discov. Today 19 (6), 743–753. 10.1016/j.drudis.2014.03.024 24704459

[B15] Brancato V. Comunanza V. Imparato G. Corà D. Urciuolo F. Noghero A. (2017a). Bioengineered tumoral microtissues recapitulate desmoplastic reaction of pancreatic cancer. Acta Biomater. 49, 152–166. 10.1016/j.actbio.2016.11.072 27916739

[B16] Brancato V. Garziano A. Gioiella F. Urciuolo F. Imparato G. Panzetta V. (2017b). 3D is not enough: Building up a cell instructive microenvironment for tumoral stroma microtissues. Acta Biomater. 47, 1–13. 10.1016/j.actbio.2016.10.007 27721010

[B17] Braziulis E. Diezi M. Biedermann T. Pontiggia L. Schmucki M. Hartmann-Fritsch F. (2012). Modified plastic compression of collagen hydrogels provides an ideal matrix for clinically applicable skin substitutes. Tissue Eng. Part C Methods 18 (6), 464–474. 10.1089/ten.tec.2011.0561 22195768

[B18] Brownfield D. G. Venugopalan G. Lo A. Mori H. Tanner K. Fletcher D. (2013). Patterned collagen fibers orient branching mammary epithelium through distinct signaling modules. Curr. Biol. 23 (8), 703–709. 10.1016/j.cub.2013.03.032 23562267 PMC3705902

[B19] Burgess J. K. Mauad T. Tjin G. Karlsson J. C. Westergren-Thorsson G. (2016). The extracellular matrix – The under-recognized element in lung disease? J. Pathol. 240 (4), 397–409. 10.1002/path.4808 27623753 PMC5129494

[B20] Burgess J. K. Muizer K. Brandsma C.-A. Heijink I. H. (2019). Dynamic reciprocity: The role of the extracellular matrix microenvironment in amplifying and sustaining pathological lung fibrosis. Berlin, Germany: Springer.

[B21] Burgstaller G. Oehrle B. Gerckens M. White E. S. Schiller H. B. Eickelberg O. (2017). The instructive extracellular matrix of the lung: Basic composition and alterations in chronic lung disease. Eur. Respir. J. 50 (1), 1601805. 10.1183/13993003.01805-2016 28679607

[B22] Burke K. Brown E. (2014). The use of second harmonic generation to image the extracellular matrix during tumor progression. IntraVital 3 (3), e984509. 10.4161/21659087.2014.984509 28243512 PMC5226008

[B23] Burke K. Tang P. Brown E. (2012). Second harmonic generation reveals matrix alterations during breast tumor progression. J. Biomed. Opt. 18 (3), 031106. 10.1117/1.jbo.18.3.031106 PMC3595714 23172133

[B24] Casale C. Imparato G. Urciuolo F. Netti P. A. (2016). Endogenous human skin equivalent promotes *in vitro* morphogenesis of follicle-like structures. Biomaterials 101, 86–95. 10.1016/j.biomaterials.2016.05.047 27267630

[B25] Casale C. Imparato G. Urciuolo F. Rescigno F. Scamardella S. Escolino M. (2018). Engineering a human skin equivalent to study dermis remodelling and epidermis senescence *in vitro* after UVA exposure. J. Tissue Eng. Regen. Med. 12 (7), 1658–1669. 10.1002/term.2693 29763974

[B26] Chen R. N. Ho H. O. Tsai Y. T. Sheu M. T. (2004). Process development of an acellular dermal matrix (ADM) for biomedical applications. Biomaterials 25 (13), 2679–2686. 10.1016/j.biomaterials.2003.09.070 14751754

[B27] Choi Y. J. Kim T. G. Jeong J. Yi H. G. Park J. W. Hwang W. (2016). 3D cell printing of functional skeletal muscle constructs using skeletal muscle-derived bioink. Adv. Healthc. Mat. 5 (20), 2636–2645. 10.1002/adhm.201600483 27529631

[B28] Choudhury D. Tun H. W. Wang T. Naing M. W. (2018). Organ-derived decellularized extracellular matrix: A game changer for bioink manufacturing? Trends Biotechnol. 36 (8), 787–805. 10.1016/j.tibtech.2018.03.003 29678431

[B29] Coates D. R. Chin J. M. Chung S. T. L. (2011b). Functional differentiation and alveolar morphogenesis of primary mammary cultures on reconstituted basement membrane. Bone 23 (1), 1–7. 10.1242/dev.105.2.223 PMC2948482 2806122

[B30] Coates D. R. Chin J. M. Chung S. T. L. (2011a). Tissue architecture and function: Dynamic reciprocity via extra- and intra-cellular matrices. Bone 23 (1), 1–7. 10.1007/s10555-008-9178-z PMC2720096 19160017

[B31] Conklin M. W. Gangnon R. E. Sprague B. L. Van Gemert L. Hampton J. M. Eliceiri K. W. (2018). Collagen alignment as a predictor of recurrence after ductal carcinoma *in situ* . Cancer Epidemiol. Biomarkers Prev. 27 (2), 138–145. 10.1158/1055-9965.epi-17-0720 29141852 PMC5809285

[B32] Corda S. Samuel J. L. Rappaport L. (2000). Extracellular matrix and growth factors during heart growth. Heart fail. Rev. 5 (2), 119–130. 10.1023/a:1009806403194 16228139

[B33] Custódio C. A. Reis R. L. Mano J. F. (2014). Engineering biomolecular microenvironments for cell instructive biomaterials. Adv. Healthc. Mat. 3 (6), 797–810. 10.1002/adhm.201300603 24464880

[B34] Das D. Noh I. (2018). Biomimetic medical materials: From nanotechnology to 3D bioprinting. Berlin, Germany: Springer.

[B35] De Gregorio V. Corrado B. Sbrescia S. Sibilio S. Urciuolo F. Netti P. A. (2020). Intestine-on-chip device increases ECM remodeling inducing faster epithelial cell differentiation. Biotechnol. Bioeng. 117 (2), 556–566. 10.1002/bit.27186 31598957

[B36] De Gregorio V. Imparato G. Urciuolo F. Tornesello M. L. Annunziata C. Buonaguro F. M. (2017). An engineered cell-instructive stroma for the fabrication of a novel full thickness human Cervix equivalent *in vitro* . Adv. Healthc. Mat. 6 (11), 1601199. 10.1002/adhm.201601199 28371541

[B37] Dede Eren A. Vermeulen S. Schmitz T. C. Foolen J. de Boer J. (2022). The loop of phenotype: Dynamic reciprocity links tenocyte morphology to tendon tissue homeostasis. Acta Biomater. 163, 275–286. 10.1016/j.actbio.2022.05.019 35584748

[B38] Del Prado-audelo M. L. Mendoza-muñoz N. Escutia-guadarrama L. Giraldo-gomez D. M. (2020). Recent advances in elastin-based biomaterials. J. Pharm. Pharm. Sci. 23, 314–332. 10.18433/jpps31254 33751927

[B39] Doyle A. D. Yamada K. M. (2016). Mechanosensing via cell-matrix adhesions in 3D microenvironments. Exp. Cell. Res. 343 (1), 60–66. 10.1016/j.yexcr.2015.10.033 26524505 PMC4851608

[B40] Drury J. L. Mooney D. J. (2003). Hydrogels for tissue engineering: Scaffold design variables and applications. Biomaterials 24, 4337–4351. 10.1016/s0142-9612(03)00340-5 12922147

[B41] Elkins R. C. Dawson P. E. Goldstein S. Walsh S. P. Black K. S. (2001). Decellularized human valve allografts. Ann. Thorac. Surg. 71 (5), S428–S432. 10.1016/s0003-4975(01)02503-6 11388241

[B42] Eltom A. Zhong G. Muhammad A. (2019). Scaffold techniques and designs in tissue engineering functions and purposes: A review. Adv. Mat. Sci. Eng. 2019, 1–13. 10.1155/2019/3429527

[B43] Emon B. Bauer J. Jain Y. Jung B. Saif T. (2018). Biophysics of tumor microenvironment and cancer metastasis - a mini review. Comput. Struct. Biotechnol. J. 16, 279–287. 10.1016/j.csbj.2018.07.003 30128085 PMC6097544

[B44] Fahimipour F. Rasoulianboroujeni M. Dashtimoghadam E. Khoshroo K. Tahriri M. Bastami F. (2017). 3D printed TCP-based scaffold incorporating VEGF-loaded PLGA microspheres for craniofacial tissue engineering. Dent. Mat. 33 (11), 1205–1216. 10.1016/j.dental.2017.06.016 PMC6013845 28882369

[B45] Fata J. E. Werb Z. Bissell M. J. (2004). Regulation of mammary gland branching morphogenesis by the extracellular matrix and its remodeling enzymes. Breast Cancer Res. 6 (1), 1–11. 10.1186/bcr634 14680479 PMC314442

[B46] Ferlin K. M. Prendergast M. E. Miller M. L. Kaplan D. S. Fisher J. P. (2016). Influence of 3D printed porous architecture on mesenchymal stem cell enrichment and differentiation. Acta Biomater. 32, 161–169. 10.1016/j.actbio.2016.01.007 26773464

[B47] Fernandez I. E. Eickelberg O. (2012). New cellular and molecular mechanisms of lung injury and fi brosis in idiopathic pulmonary fi brosis. Lancet 380 (9842), 680–688. 10.1016/s0140-6736(12)61144-1 22901889

[B48] Martino F. Perestrelo A. R. Vinarský V. Pagliari S. Forte G. (2018). “Cellular mechanotransduction: From tension to function,” Front. Physiol. 9, 824–921. 10.3389/fphys.2018.00824 30026699 PMC6041413

[B49] Frantz C. Stewart K. M. Weaver V. M. (2010). The extracellular matrix at a glance. J. Cell. Sci. 123 (24), 4195–4200. 10.1242/jcs.023820 21123617 PMC2995612

[B50] Gao G. Lee J. H. Jang J. Lee D. H. Kong J. S. Kim B. S. (2017). Tissue engineered bio-blood-vessels constructed using a tissue-specific bioink and 3D coaxial cell printing technique: A novel therapy for ischemic disease. Adv. Funct. Mat. 27 (33), 1700798–1700812. 10.1002/adfm.201700798

[B51] Gharzi A. Reynolds A. J. Jahoda C. A. B. (2003). Plasticity of hair follicle dermal cells in wound healing and induction. Exp. Dermatol. 12 (2), 126–136. 10.1034/j.1600-0625.2003.00106.x 12702140

[B52] Gioiella F. Urciuolo F. Imparato G. Brancato V. Netti P. A. (2016). An engineered breast cancer model on a chip to replicate ECM-activation *in vitro* during tumor progression. Adv. Healthc. Mat. 5 (23), 3074–3084. 10.1002/adhm.201600772 27925458

[B53] Gjorevski N. Ranga A. Lutolf M. P. (2014). Bioengineering approaches to guide stem cell-based organogenesis. Dev 141 (9), 1794–1804. 10.1242/dev.101048 24757002

[B54] Godwin A. R. F. Singh M. Lockhart-Cairns M. P. Alanazi Y. F. Cain S. A. Baldock C. (2019). The role of fibrillin and microfibril binding proteins in elastin and elastic fibre assembly. Matrix Biol. 84, 17–30. 10.1016/j.matbio.2019.06.006 31226403 PMC6943813

[B55] Guaccio A. Borselli C. Oliviero O. Netti P. A. (2008). Oxygen consumption of chondrocytes in agarose and collagen gels: A comparative analysis. Biomaterials 29 (10), 1484–1493. 10.1016/j.biomaterials.2007.12.020 18191194

[B56] Guarino V. Urciuolo F. Alvarez-Perez M. A. Mele B. Netti P. A. Ambrosio L. (2012). Osteogenic differentiation and mineralization in fibre-reinforced tubular scaffolds: Theoretical study and experimental evidences. J. R. Soc. Interface 9 (74), 2201–2212. 10.1098/rsif.2011.0913 22399788 PMC3405741

[B57] Guddati S. Kiran A. S. K. Leavy M. Ramakrishna S. (2019). Recent advancements in additive manufacturing technologies for porous material applications. Int. J. Adv. Manuf. Technol. 105 (1–4), 193–215. 10.1007/s00170-019-04116-z

[B58] Guneta V. Loh Q. L. Choong C. (2016). Cell-secreted extracellular matrix formation and differentiation of adipose-derived stem cells in 3D alginate scaffolds with tunable properties. J. Biomed. Mat. Res. - Part A 104 (5), 1090–1101. 10.1002/jbm.a.35644 26749566

[B59] Gupta P. Pérez-Mancera P. A. Kocher H. Nisbet A. Schettino G. Velliou E. G. (2020). A novel scaffold-based hybrid multicellular model for pancreatic ductal adenocarcinoma—toward a better mimicry of the *in vivo* tumor microenvironment. Front. Bioeng. Biotechnol. 8, 290. 10.3389/fbioe.2020.00290 32391339 PMC7193232

[B60] Hallgren O. Nihlberg K. Dahlbäck M. Bjermer L. Eriksson L. T. Erjefält J. S. (2010). Altered fibroblast proteoglycan production in COPD. Respir. Res. 11, 55–11. 10.1186/1465-9921-11-55 20459817 PMC2886021

[B61] Halstenberg S. Panitch A. Rizzi S. Hall H. Hubbell J. A. (2002). Biologically engineered protein-graft-poly(ethylene glycol) hydrogels: A cell adhesive and plasmin-degradable biosynthetic material for tissue repair. Biomacromolecules 3 (4), 710–723. 10.1021/bm015629o 12099815

[B62] Han W. Chen S. Yuan W. Fan Q. Tian J. Wang X. (2016). Oriented collagen fibers direct tumor cell intravasation. Proc. Natl. Acad. Sci. U. S. A. 113 (40), 11208–11213. 10.1073/pnas.1610347113 27663743 PMC5056065

[B63] Haring A. P. Thompson E. G. Tong Y. Laheri S. Cesewski E. Sontheimer H. (2019). Process- and bio-inspired hydrogels for 3D bioprinting of soft free-standing neural and glial tissues. Biofabrication 11 (2), 025009. 10.1088/1758-5090/ab02c9 30695770

[B64] Harris A. K. Wild P. Stopak D. (1980). Silicone rubber substrata: A new wrinkle in the study of cell locomotion. Science 208 (4440), 177–179. 10.1126/science.6987736 6987736

[B65] Hennessy K. M. Pollot B. E. Clem W. C. Phipps M. C. Sawyer A. A. Culpepper B. K. (2009). The effect of collagen I mimetic peptides on mesenchymal stem cell adhesion and differentiation, and on bone formation at hydroxyapatite surfaces. Biomaterials 30 (10), 1898–1909. 10.1016/j.biomaterials.2008.12.053 19157536 PMC3679919

[B66] Higuchi Y. Shiraki N. Yamane K. Qin Z. Mochitate K. Araki K. (2010). Synthesized basement membranes direct the differentiation of mouse embryonic stem cells into pancreatic lineages. J. Cell. Sci. 123 (16), 2733–2742. 10.1242/jcs.066886 20647375

[B67] Hinek A. Kim H. J. Wang Y. Wang A. Mitts T. F. (2014). Sodium l-ascorbate enhances elastic fibers deposition by fibroblasts from normal and pathologic human skin. J. Dermatol. Sci. 75 (3), 173–182. 10.1016/j.jdermsci.2014.05.011 25015208

[B68] Hoshiba T. (2018). An extracellular matrix (ECM) model at high malignant colorectal tumor increases chondroitin sulfate chains to promote epithelial-mesenchymal transition and chemoresistance acquisition. Exp. Cell. Res. 370 (2), 571–578. 10.1016/j.yexcr.2018.07.022 30016638

[B69] Hoshiba T. Tanaka M. (2013). Breast cancer cell behaviors on staged tumorigenesis-mimicking matrices derived from tumor cells at various malignant stages. Biochem. Biophys. Res. Commun. 439 (2), 291–296. 10.1016/j.bbrc.2013.08.038 23978418

[B70] Hosoyama K. Lazurko C. Muñoz M. McTiernan C. D. Alarcon E. I. (2019). Peptide-based functional biomaterials for soft-tissue repair. Front. Bioeng. Biotechnol. 7, 205. 10.3389/fbioe.2019.00205 31508416 PMC6716508

[B71] Hu M. Polyak K. (2008). Microenvironmental regulation of cancer development. Curr. Opin. Genet. Dev. 18 (1), 27–34. 10.1016/j.gde.2007.12.006 18282701 PMC2467152

[B72] Hutmacher D. W. Sittinger M. Risbud M. V. (2004). Scaffold-based tissue engineering: Rationale for computer-aided design and solid free-form fabrication systems. Trends Biotechnol. 22 (7), 354–362. 10.1016/j.tibtech.2004.05.005 15245908

[B73] Hynes R. O. Destree A. T. Perkins M. E. Wagner D. D. (1979). Cell surface fibronectin and oncogenic transformation. J. Supramol. Struct. 11 (1), 95–104. 10.1002/jss.400110110 522486

[B74] Hynes R. O. (2012). The evolution of metazoan extracellular matrix. J. Cell. Biol. 196 (6), 671–679. 10.1083/jcb.201109041 22431747 PMC3308698

[B75] Iannone M. Ventre M. Formisano L. Casalino L. Patriarca E. J. Netti P. A. (2015). Nanoengineered surfaces for focal adhesion guidance trigger mesenchymal stem cell self-organization and tenogenesis. Nano Lett. 15 (3), 1517–1525. 10.1021/nl503737k 25699511

[B76] Imparato G. Casale C. Scamardella S. Urciuolo F. Bimonte M. Apone F. (2017). A novel engineered dermis for *in vitro* photodamage research. J. Tissue Eng. Regen. Med. 11 (8), 2276–2285. 10.1002/term.2125 26857337

[B77] Imparato G. Urciuolo F. Casale C. Netti P. A. Imparato P. A. Urciuolo F. (2013). The role of microscaffold properties in controlling the collagen assembly in 3D dermis equivalent using modular tissue engineering. Biomat 34 (32), 7851–7861. 10.1016/j.biomaterials.2013.06.062 23891518

[B78] Ishida S. (2018). Organs-on-a-chip: Current applications and consideration points for *in vitro* ADME-Tox studies. Drug Metab. Pharmacokinet. 33 (1), 49–54. 10.1016/j.dmpk.2018.01.003 29398302

[B79] Faralli J. A. Filla M. S. Peters D. M. (2022). “Integrin crosstalk and its effect on the biological functions of the trabecular meshwork/schlemm’s canal,” Front. Cell. Dev. Biol. 10, 886702–886709. 10.3389/fcell.2022.886702 35573686 PMC9099149

[B80] Jahed Z. Shams H. Mehrbod M. Mofrad M. R. K. (2014). Mechanotransduction pathways linking the extracellular matrix to the nucleus. Int. Rev. Cell. Mol. Biol. 310, 171–220. 10.1016/B978-0-12-800180-6.00005-0 24725427

[B81] Nichols J. E. Niles J. Riddle M. Vargas G. Schilagard T. Ma L. (2013). Production and assessment of decellularized pig and human lung scaffolds,” Tissue Eng. - Part A 19, 2045–2062. 10.1089/ten.tea.2012.0250 23638920 PMC3725800

[B82] Meng J. Wang G. Zhou L. Jiang S. Qian S. Chen L. (2023). Mapping variation of extracellular matrix in human keloid scar by label-free multiphoton imaging and machine learning,” J. Biomed. Opt. 28, 1–14. 10.1117/1.jbo.28.4.045001 PMC10082605 37038546

[B83] Johnson P. R. A. Burgess J. K. Ge Q. Poniris M. Boustany S. Twigg S. M. (2006). Connective tissue growth factor induces extracellular matrix in asthmatic airway smooth muscle. Am. J. Respir. Crit. Care Med. 173 (1), 32–41. 10.1164/rccm.200406-703oc 16179645

[B84] Kanchanawong P. Calderwood D. A. (2023). Organization, dynamics and mechanoregulation of integrin-mediated cell–ECM adhesions. Nat. Rev. Mol. Cell. Biol. 24 (2), 142–161. 10.1038/s41580-022-00531-5 36168065 PMC9892292

[B85] Karamanos N. K. Theocharis A. D. Piperigkou Z. Manou D. Passi A. Skandalis S. S. (2021). A guide to the composition and functions of the extracellular matrix. FEBS J. 288 (24), 6850–6912. 10.1111/febs.15776 33605520

[B86] Kaukonen R. Jacquemet G. Hamidi H. Ivaska J. (2017). Cell-derived matrices for studying cell proliferation and directional migration in a complex 3D microenvironment. Nat. Protoc. 12 (11), 2376–2390. 10.1038/nprot.2017.107 29048422

[B87] Kilmer P. D. (2010). Review article: Review article: Doug underwood journalism and the novel: Truth and fiction, 1700—2000. Journalism 11 (3), 369–373. 10.1177/1461444810365020

[B88] Kim H. K. Kim J. H. Park D. S. Park K. S. Kang S. S. Lee J. S. (2012). Osteogenesis induced by a bone forming peptide from the prodomain region of BMP-7. Biomaterials 33 (29), 7057–7063. 10.1016/j.biomaterials.2012.06.036 22795855

[B89] Kim S. E. Lee P. W. Pokorski J. K. (2017). Biologically triggered delivery of EGF from polymer fiber patches. ACS Macro Lett. 6 (6), 593–597. 10.1021/acsmacrolett.7b00212 29250460 PMC5726586

[B90] Kishimoto J. Ehama R. Wu L. Jiang S. Jiang N. Burgeson R. E. (1999). Selective activation of the versican promoter by epithelial-mesenchymal interactions during hair follicle development. Proc. Natl. Acad. Sci. U. S. A. 96 (13), 7336–7341. 10.1073/pnas.96.13.7336 10377415 PMC22086

[B91] Kleinman H. K. McGarvey M. L. Hassell J. R. Star V. L. Cannon F. B. Laurie G. W. (1986). Basement membrane complexes with biological activity. Biochemistry 25 (2), 312–318. 10.1021/bi00350a005 2937447

[B92] Klimek K. Ginalska G. (2020). Proteins and peptides as important modifiers of the polymer scaffolds for tissue engineering applications-A review. Polym. (Basel) 12 (4), 844. 10.3390/polym12040844 PMC7240665 32268607

[B93] Knudson W. Peterson R. S. (2004). The hyaluronan receptor: CD44. Chem. Biol. Hyaluronan 298, 83–123.

[B94] Korosec A. Lichtenberger B. M. (2017). *In vitro* models to study hair follicle generation. Netherlands: Elsevier.

[B95] Krishna O. D. Kiick K. L. (2010). Protein- and peptide-modified synthetic polymeric biomaterials. Biopolymers 94 (1), 32–48. 10.1002/bip.21333 20091878 PMC4437713

[B96] Kuboki Y. Jin Q. Takita H. (2001). Geometry of carriers controlling phenotypic expression in BMP-induced osteogenesis and chondrogenesis. J. Bone Jt. Surg. 83, 105–115. 10.2106/00004623-200100002-00005 11314788

[B97] Kubow K. E. Klotzsch E. Smith M. L. Gourdon D. Little W. C. Vogel V. (2009). Crosslinking of cell-derived 3D scaffolds up-regulates the stretching and unfolding of new extracellular matrix assembled by reseeded cells. Integr. Biol. 1 (11–12), 635–648. 10.1039/b914996a PMC3818580 20027372

[B98] Kunz-Schughart L. A. Knuechel R. (2002). Tumor-associated fibroblasts (Part I): Active stromal participants in tumor development and progression? Histol. Histopathol. 17 (2), 599–621. 10.14670/HH-17.599 11962761

[B99] Langhans S. A. (2018). Three-dimensional *in vitro* cell culture models in drug discovery and drug repositioning. Front. Pharmacol. 9, 6–14. 10.3389/fphar.2018.00006 29410625 PMC5787088

[B100] LaPolt P. S. Lu J. K. H. (2001). Effects of aging on luteinizing hormone secretion, ovulation, and ovarian tissue-type plasminogen activator expression. Proc. Soc. Exp. Biol. Med. 226 (2), 127–132. 10.1177/153537020122600210 11446436

[B101] Larsen K. Tufvesson E. Malmström J. Mörgelin M. Wildt M. Andersson A. (2004). Presence of activated mobile fibroblasts in bronchoalveolar lavage from patients with mild asthma. Am. J. Respir. Crit. Care Med. 170 (10), 1049–1056. 10.1164/rccm.200404-507oc 15256392

[B102] Larsen N. P. Paddock R. Alexander H. L. (2015). “Bronchial asthma and allied conditions: Clinical and immunological observations,” J. Immunol. 7, 81–95. 13759191

[B103] Lee J. Rabbani C. C. Gao H. Steinhart M. R. Woodruff B. M. Pflum Z. E. (2020). Hair-bearing human skin generated entirely from pluripotent stem cells. Nature 582 (7812), 399–404. 10.1038/s41586-020-2352-3 32494013 PMC7593871

[B104] Lee K. Y. Mooney D. J. (2001). Hydrogels for tissue engineering. Chem. Rev. 101 (7), 1869–1880. 10.1021/cr000108x 11710233

[B105] Lien S. M. Ko L. Y. Huang T. J. (2009). Effect of pore size on ECM secretion and cell growth in gelatin scaffold for articular cartilage tissue engineering. Acta Biomater. 5 (2), 670–679. 10.1016/j.actbio.2008.09.020 18951858

[B106] Lin Z. Y. Duan Z. X. Guo X. D. Li J. F. Lu H. W. Zheng Q. X. (2010). Bone induction by biomimetic PLGA-(PEG-ASP)n copolymer loaded with a novel synthetic BMP-2-related peptide *in vitro* and *in vivo* . J. Control. Release 144 (2), 190–195. 10.1016/j.jconrel.2010.02.016 20184932

[B107] Liu C. Chiang B. Lewin Mejia D. Luker K. E. Luker G. D. Lee A. (2019). Mammary fibroblasts remodel fibrillar collagen microstructure in a biomimetic nanocomposite hydrogel. Acta Biomater. 83, 221–232. 10.1016/j.actbio.2018.11.010 30414485 PMC6291359

[B108] Liu K. Cheng L. Flesken-Nikitin A. Huang L. Nikitin A. Y. Pauli B. U. (2010). Conditional knockout of fibronectin abrogates mouse mammary gland lobuloalveolar differentiation. Dev. Biol. 346 (1), 11–24. 10.1016/j.ydbio.2010.07.001 20624380 PMC2937099

[B109] Loh Q. L. Choong C. (2013). Three-dimensional scaffolds for tissue engineering applications: Role of porosity and pore size. Tissue Eng. - Part B Rev. 19 (6), 485–502. 10.1089/ten.teb.2012.0437 23672709 PMC3826579

[B110] Lombardi B. Casale C. Imparato G. Urciuolo F. Netti P. A. (2017). Spatiotemporal evolution of the wound repairing process in a 3D human dermis equivalent. Adv. Healthc. Mat. 6 (13), 1601422–1601511. 10.1002/adhm.201601422 28407433

[B111] Lut M. P. Tirelli N. Cerritelli S. Cavalli L. Hubbell J. A. (2001). Systematic modulation of michael - type reactivity of thiols through the use of charged amino acids. Bioconjug Chem. 12, 1051–1056. 10.1021/bc015519e 11716699

[B112] Lutolf M. P. Hubbell J. A. (2005). Synthetic biomaterials as instructive extracellular microenvironments for morphogenesis in tissue engineering. Nat. Biotechnol. 23 (1), 47–55. 10.1038/nbt1055 15637621

[B113] Lutolf M. P. Lauer-Fields J. L. Schmoekel H. G. Metters A. T. Weber F. E. Fields G. B. (2003). Synthetic matrix metalloproteinase-sensitive hydrogels for the conduction of tissue regeneration: Engineering cell-invasion characteristics. Proc. Natl. Acad. Sci. U. S. A. 100 (9), 5413–5418. 10.1073/pnas.0737381100 12686696 PMC154359

[B114] Malik R. Lelkes P. I. Cukierman E. (2015). Biomechanical and biochemical remodeling of stromal extracellular matrix in cancer. Trends Biotechnol. 33 (4), 230–236. 10.1016/j.tibtech.2015.01.004 25708906 PMC4380578

[B115] Mandal B. B. Kundu S. C. (2009). Cell proliferation and migration in silk fibroin 3D scaffolds. Biomaterials 30 (15), 2956–2965. 10.1016/j.biomaterials.2009.02.006 19249094

[B116] Martin I. Wendt D. Heberer M. (2004). The role of bioreactors in tissue engineering. Trends Biotechnol. 22 (2), 80–86. 10.1016/j.tibtech.2003.12.001 14757042

[B117] Matsiko A. Gleeson J. P. Bai B. A. Brien F. J. O. (2015). Scaffold mean pore size influences mesenchymal stem cell chondrogenic differentiation and matrix deposition Authors Tissue Engineering Research Group. Tissue Eng. Part A 21, 486–497. 10.1089/ten.TEA.2013.0545 25203687

[B118] Mazio C. Casale C. Imparato G. Urciuolo F. Netti P. A. (2018). Recapitulating spatiotemporal tumor heterogeneity *in vitro* through engineered breast cancer microtissues. Acta Biomater. 73, 236–249. 10.1016/j.actbio.2018.04.028 29679778

[B119] Mazza G. Rombouts K. Rennie Hall A. Urbani L. Vinh Luong T. Al-Akkad W. (2015). Decellularized human liver as a natural 3D-scaffold for liver bioengineering and transplantation. Sci. Rep. 5, 13079–13115. 10.1038/srep13079 26248878 PMC4528226

[B120] Miller A. E. Hu P. Barker T. H. (2020). Feeling things out: Bidirectional signaling of the cell–ECM interface, implications in the mechanobiology of cell spreading, migration, proliferation, and differentiation. Adv. Healthc. Mat. 9 (8), 1901445–1901524. 10.1002/adhm.201901445 PMC7274903 32037719

[B121] Muncie J. M. Ayad N. M. E. Lakins J. N. Xue X. Fu J. Weaver V. M. (2020). Mechanical tension promotes formation of gastrulation-like nodes and patterns mesoderm specification in human embryonic stem cells. Dev. Cell. 55 (6), 679–694.e11. 10.1016/j.devcel.2020.10.015 33207224 PMC7755684

[B122] Münster S. Jawerth L. M. Leslie B. A. Weitz J. I. Fabry B. Weitz D. A. (2013). Strain history dependence of the nonlinear stress response of fibrin and collagen networks. Proc. Natl. Acad. Sci. U. S. A. 110 (30), 12197–12202. 10.1073/pnas.1222787110 23754380 PMC3725119

[B123] Nelson C. M. Bissell M. J. Division L. S. Berkeley L. (2006). Of extracellular matrix, scaffolds, and signaling: Tissue architecture regulates development, homeostasis, and cancer. Annu. Rev. Cell. Dev. Biol. 22, 287–309. 10.1146/annurev.cellbio.22.010305.104315 16824016 PMC2933192

[B124] Nemec S. Kilian K. A. (2021). Materials control of the epigenetics underlying cell plasticity. Nat. Rev. Mat. 6 (1), 69–83. 10.1038/s41578-020-00238-z

[B125] Netti P. (2019). Bioactivated materials for cell and tissue guidance. Berlin, Germany: Springer.

[B126] Orlando G. Booth C. Wang Z. Totonelli G. Ross C. L. Moran E. (2013). Discarded human kidneys as a source of ECM scaffold for kidney regeneration technologies. Biomaterials 34 (24), 5915–5925. 10.1016/j.biomaterials.2013.04.033 23680364

[B127] Ott H. C. Clippinger B. Conrad C. Schuetz C. Pomerantseva I. Ikonomou L. (2010). Regeneration and orthotopic transplantation of a bioartificial lung. Nat. Med. 16 (8), 927–933. 10.1038/nm.2193 20628374

[B128] Parenteau-Bareil R. Gauvin R. Berthod F. (2010). Collagen-based biomaterials for tissue engineering applications. Mater. (Basel) 3 (3), 1863–1887. 10.3390/ma3031863

[B129] Pati F. Jang J. Ha D. H. Won Kim S. Rhie J. W. Shim J. H. (2014). Printing three-dimensional tissue analogues with decellularized extracellular matrix bioink. Nat. Commun. 5, 3935. 10.1038/ncomms4935 24887553 PMC4059935

[B130] Patra S. Young V. (2016). A review of 3D printing techniques and the future in biofabrication of bioprinted tissue. Cell. biochem. Biophys. 74 (2), 93–98. 10.1007/s12013-016-0730-0 27193609

[B131] Pelham R. J. Wang Y. L. (1997). Cell locomotion and focal adhesions are regulated by substrate flexibility. Proc. Natl. Acad. Sci. U. S. A. 94 (25), 13661–13665. 10.1073/pnas.94.25.13661 9391082 PMC28362

[B132] Pereira D. Richert A. Medjkane S. Hénon S. Weitzman J. B. (2020). Cell geometry and the cytoskeleton impact the nucleo-cytoplasmic localisation of the SMYD3 methyltransferase. Sci. Rep. 10 (1), 20598–20612. 10.1038/s41598-020-75833-9 33244033 PMC7691988

[B133] Pountos I. Panteli M. Lampropoulos A. Jones E. Calori G. M. Giannoudis P. V. (2016). The role of peptides in bone healing and regeneration: A systematic review. BMC Med. 14 (1), 103–115. 10.1186/s12916-016-0646-y 27400961 PMC4940902

[B134] Qu M. Jiang X. Zhou X. Wang C. Wu Q. Ren L. (2020). Stimuli-responsive delivery of growth factors for tissue engineering. Adv. Healthc. Mat. 9 (7), 1901714–1901719. 10.1002/adhm.201901714 PMC7189772 32125786

[B135] Ragelle H. Naba A. Larson B. L. Zhou F. Prijić M. Whittaker C. A. (2017). Comprehensive proteomic characterization of stem cell-derived extracellular matrices. Biomaterials 128, 147–159. 10.1016/j.biomaterials.2017.03.008 28327460 PMC8191742

[B136] Rajab T. K. O’Malley T. J. Tchantchaleishvili V. (2020). Decellularized scaffolds for tissue engineering: Current status and future perspective. Artif. Organs 44 (10), 1031–1043. 10.1111/aor.13701 32279344

[B137] Range K. D. M. Moser Y. A. (2012). Extracellular matrix signaling in morphogenesis and repair. Bone 23 (1), 1–7.

[B138] Rittié L. Fisher G. J. (2015). Natural and sun-induced aging of human skin. Cold Spring Harb. Perspect. Med. 5 (1), a015370. 10.1101/cshperspect.a015370 25561721 PMC4292080

[B139] Rnjak-Kovacina J. Wise S. G. Li Z. Maitz P. K. Young C. J. Wang Y. (2011). Tailoring the porosity and pore size of electrospun synthetic human elastin scaffolds for dermal tissue engineering. Biomaterials 32 (28), 6729–6736. 10.1016/j.biomaterials.2011.05.065 21683438

[B140] Roy V. Magne B. Vaillancourt-Audet M. Blais M. Chabaud S. Grammond E. (2020). Human organ-specific 3D cancer models produced by the stromal self-assembly method of tissue engineering for the study of solid tumors. Biomed. Res. Int. 2020, 1–23. 10.1155/2020/6051210 PMC7178531 32352002

[B141] Rozario T. DeSimone D. W. (2010). The extracellular matrix in development and morphogenesis: A dynamic view. Dev. Biol. 341 (1), 126–140. 10.1016/j.ydbio.2009.10.026 19854168 PMC2854274

[B142] Rybinski B. Franco-Barraza J. Cukierman E. (2014). The wound healing, chronic fibrosis, and cancer progression triad. Physiol. Genomics 46 (7), 223–244. 10.1152/physiolgenomics.00158.2013 24520152 PMC4035661

[B143] Sainio A. Järveläinen H. (2020). “Extracellular matrix-cell interactions: Focus on therapeutic applications,” Cell. Signal. 66, 109487. 10.1016/j.cellsig.2019.109487 31778739

[B144] Sakar M. S. Eyckmans J. Pieters R. Eberli D. Nelson B. J. Chen C. S. (2016). Cellular forces and matrix assembly coordinate fibrous tissue repair. Nat. Commun. 7, 11036. 10.1038/ncomms11036 26980715 PMC4799373

[B145] Saludas L. Pascual-Gil S. Prósper F. Garbayo E. Blanco-Prieto M. (2017). Hydrogel based approaches for cardiac tissue engineering. Int. J. Pharm. 523 (2), 454–475. 10.1016/j.ijpharm.2016.10.061 27989830

[B146] Saska S. Pilatti L. Blay A. Shibli J. A. (2021). Bioresorbable polymers: Advanced materials and 4D printing for tissue engineering. Polym. (Basel) 13 (4), 563–624. 10.3390/polym13040563 PMC7918883 33668617

[B147] Satyam A. Tsokos M. G. Tresback J. S. Zeugolis D. I. Tsokos G. C. (2020). Cell-derived extracellular matrix-rich biomimetic substrate supports podocyte proliferation, differentiation, and maintenance of native phenotype. Adv. Funct. Mat. 30 (44), 1908752–1908811. 10.1002/adfm.201908752 PMC7939063 33692659

[B148] Schmidt C. E. Baier J. M. (2000). Acellular vascular tissues: Natural biomaterials for tissue repair and tissue engineering. Biomaterials 21 (22), 2215–2231. 10.1016/s0142-9612(00)00148-4 11026628

[B149] Schultheiss D. Gabouev A. I. Cebotari S. Tudorache I. Walles T. Schlote N. (2005). Biological vascularized matrix for bladder tissue engineering: Matrix preparation, reseeding technique and short-term implantation in a porcine model. J. Urol. 173 (1), 276–280. 10.1097/01.ju.0000145882.80339.18 15592096

[B150] Selman M. Pardo A. (2021). Fibroageing: An ageing pathological feature driven by dysregulated extracellular matrix-cell mechanobiology. Ageing Res. Rev. 70, 101393. 10.1016/j.arr.2021.101393 34139337

[B151] Shi M. Zhang H. Song T. Liu X. Gao Y. Zhou J. (2019). Sustainable dual release of antibiotic and growth factor from pH-responsive uniform alginate composite microparticles to enhance wound healing. ACS Appl. Mat. Interfaces 11, 22730–22744. 10.1021/acsami.9b04750 31141337

[B152] Shinsato Y. Doyle A. D. Li W. Yamada K. M. (2020). Direct comparison of five different 3D extracellular matrix model systems for characterization of cancer cell migration. Cancer Rep. 3 (5), 12577–e1311. 10.1002/cnr2.1257 PMC7941507 33085847

[B153] Silberstein G. B. Daniel C. W. (1984). Glycosaminoglycans in the basal lamina and extracellular matrix of serially aged mouse mammary ducts. Mech. Ageing Dev. 24 (2), 151–162. 10.1016/0047-6374(84)90067-8 6717086

[B154] Skardal A. Devarasetty M. Kang H. W. Mead I. Bishop C. Shupe T. (2015). A hydrogel bioink toolkit for mimicking native tissue biochemical and mechanical properties in bioprinted tissue constructs. Acta Biomater. 25, 24–34. 10.1016/j.actbio.2015.07.030 26210285

[B155] Solarte David V. A. Güiza-Argüello V. R. Arango-Rodríguez M. L. Sossa C. L. Becerra-Bayona S. M. (2022). Decellularized tissues for wound healing: Towards closing the gap between scaffold design and effective extracellular matrix remodeling. Front. Bioeng. Biotechnol. 10, 821852–821926. 10.3389/fbioe.2022.821852 35252131 PMC8896438

[B156] Stahl P. J. Chan T. R. Shen Y. I. Sun G. Gerecht S. Yu S. M. (2014). Capillary network-like organization of endothelial cells in PEGDA scaffolds encoded with angiogenic signals via triple helical hybridization. Adv. Funct. Mat. 24 (21), 3213–3225. 10.1002/adfm.201303217 PMC4273917 25541582

[B157] Subbiah R. Guldberg R. E. (2019). Materials science and design principles of growth factor delivery systems in tissue engineering and regenerative medicine. Adv. Healthc. Mat. 8 (1), 1801000–1801024. 10.1002/adhm.201801000 30398700

[B158] Subbiah R. Hipfinger C. Tahayeri A. Athirasala A. Horsophonphong S. Thrivikraman G. (2020). 3D printing of Microgel-loaded Modular LEGO-like Cages as Instructive Scaffolds for Tissue Engineering TOC 3D printed LEGO-like hollow microcages can be easily assembled, adjoined, and stacked-up to suit the complexity of defect tissues; aid spatial lo. Adv. Mater 32, e2001736. 10.1002/adma.202001736 32700332

[B159] Swanson W. B. (2022). Scaffold pore curvature influences MSC fate through differential cellular organization and YAP/TAZ activity. Int. J. Mol. Sci. 23 (9), 1–27. 10.3390/ijms23094499 PMC9102667 35562890

[B160] Swinehart I. T. Badylak S. F. (2016). Extracellular matrix bioscaffolds in tissue remodeling and morphogenesis. Dev. Dyn. 245 (3), 351–360. 10.1002/dvdy.24379 26699796 PMC4755921

[B161] Takahashi S. Yamazoe H. Sassa F. Suzuki H. Fukuda J. (2009). Preparation of coculture system with three extracellular matrices using capillary force lithography and layer-by-layer deposition. J. Biosci. Bioeng. 108 (6), 544–550. 10.1016/j.jbiosc.2009.06.013 19914591

[B162] Takahashi Y. Tabata Y. (2004). Effect of the fiber diameter and porosity of non-woven PET fabrics on the osteogenic differentiation of mesenchymal stem cells. J. Biomater. Sci. Polym. Ed. 15 (1), 41–57. 10.1163/156856204322752228 15027842

[B163] Taniguchi N. Fujibayashi S. Takemoto M. Sasaki K. Otsuki B. Nakamura T. (2016). Effect of pore size on bone ingrowth into porous titanium implants fabricated by additive manufacturing: An *in vivo* experiment. Mat. Sci. Eng. C 59, 690–701. 10.1016/j.msec.2015.10.069 26652423

[B164] Tarafder S. Koch A. Jun Y. Chou C. Awadallah M. R. Lee C. H. (2016). Micro-precise spatiotemporal delivery system embedded in 3D printing for complex tissue regeneration. Biofabrication 8 (2), 025003. 10.1088/1758-5090/8/2/025003 27108484

[B165] Taufalele P. V. VanderBurgh J. A. Muñoz A. Zanotelli M. R. Reinhart-King C. A. (2019). Fiber alignment drives changes in architectural and mechanical features in collagen matrices. PLoS One 14 (5), 02165377–e216611. 10.1371/journal.pone.0216537 PMC6519824 31091287

[B166] Thompson D. M. Buettner H. M. (2006). Neurite outgrowth is directed by schwann cell alignment in the absence of other guidance cues. Ann. Biomed. Eng. 34 (4), 161–168. 10.1007/s10439-005-9013-4 16453203

[B167] Thorne J. T. Segal T. R. Chang S. Jorge S. Segars J. H. Leppert P. C. (2015). Dynamic reciprocity between cells and their microenvironment in reproduction. Biol. Reprod. 92 (1), 25–10. 10.1095/biolreprod.114.121368 25411389 PMC4434933

[B168] Totaro A. Urciuolo F. Imparato G. Netti P. A. (2016). Engineered cardiac micromodules for the *in vitro* fabrication of 3D endogenous macro-tissues. Biofabrication 8 (2), 025014. 10.1088/1758-5090/8/2/025014 27213995

[B169] Turley E. A. Austen L. Vandeligt K. Clary C. (1991). Hyaluronan and a cell-associated hyaluronan binding protein regulate the locomotion of ras-transformed cells. J. Cell. Biol. 112 (5), 1041–1047. 10.1083/jcb.112.5.1041 1705559 PMC2288867

[B170] Urciuolo F. Garziano A. Imparato G. Panzetta V. Fusco S. Casale C. (2016). Biophysical properties of dermal building-blocks affect extra cellular matrix assembly in 3D endogenous macrotissue. Biofabrication 8 (1), 015010. 10.1088/1758-5090/8/1/015010 26824879

[B171] Urciuolo F. Imparato G. Palmiero C. Trilli A. Netti P. A. (2011). Effect of process conditions on the growth of three-dimensional dermal-equivalent tissue obtained by microtissue precursor assembly. Tissue Eng. - Part C Methods 17 (2), 155–164. 10.1089/ten.tec.2010.0355 20704470

[B172] Urciuolo F. Passariello R. Imparato G. Casale C. Netti P. A. (2022). Bioengineered wound healing skin models: The role of immune response and endogenous ECM to fully replicate the dynamic of scar tissue formation *in vitro* . Bioengineering 9 (6), 233. 10.3390/bioengineering9060233 35735476 PMC9219817

[B173] Ventre M. Causa F. Netti P. A. (2012). Determinants of cell-material crosstalk at the interface: Towards engineering of cell instructive materials. J. R. Soc. Interface 9 (74), 2017–2032. 10.1098/rsif.2012.0308 22753785 PMC3405766

[B174] Vrana N. E. Elsheikh A. Builles N. Damour O. Hasirci V. (2007). Effect of human corneal keratocytes and retinal pigment epithelial cells on the mechanical properties of micropatterned collagen films. Biomaterials 28 (29), 4303–4310. 10.1016/j.biomaterials.2007.06.013 17618681

[B175] Wang Z. Wang L. Li T. Liu S. Guo B. Huang W. (2021). 3D bioprinting in cardiac tissue engineering. Theranostics 11 (16), 7948–7969. 10.7150/thno.61621 34335973 PMC8315053

[B176] Watt F. M. (2016). Engineered microenvironments to direct epidermal stem cell behavior at single-cell resolution. Dev. Cell. 38 (6), 601–609. 10.1016/j.devcel.2016.08.010 27676433

[B177] Webber J. P. Spary L. K. Sanders A. J. Chowdhury R. Jiang W. G. Steadman R. (2015). Differentiation of tumour-promoting stromal myofibroblasts by cancer exosomes. Oncogene 34 (3), 290–302. 10.1038/onc.2013.560 24441045

[B178] Wen H. He B. Wang H. Chen F. Li P. Cui M. (2019). Structure-based gastro-retentive and controlled-release drug delivery with novel 3D printing. AAPS PharmSciTech 20 (2), 68. 10.1208/s12249-018-1237-3 30627938

[B179] Westergren-Thorsson G. Sime P. Jordana M. Gauldie J. Särnstrand B. Malmström A. (2004). Lung fibroblast clones from normal and fibrotic subjects differ in hyaluronan and decorin production and rate of proliferation. Int. J. Biochem. Cell. Biol. 36 (8), 1573–1584. 10.1016/j.biocel.2004.01.009 15147736

[B180] Wiseman B. S. Sternlicht M. D. Lund L. R. Alexander C. M. Mott J. Bissell M. J. (2003). Site-specific inductive and inhibitory activities of MMP-2 and MMP-3 orchestrate mammary gland branching morphogenesis. J. Cell. Biol. 162 (6), 1123–1133. 10.1083/jcb.200302090 12975354 PMC2172848

[B181] Wu H. Yu Y. Huang H. Hu Y. Fu S. Wang Z. (2020). Progressive pulmonary fibrosis is caused by elevated mechanical tension on alveolar stem cells. Cell. 180 (1), 107–121.e17. 10.1016/j.cell.2019.11.027 31866069

[B182] Wubneh A. Tsekoura E. K. Ayranci C. Uludağ H. (2018). Current state of fabrication technologies and materials for bone tissue engineering. Acta Biomater. 80, 1–30. 10.1016/j.actbio.2018.09.031 30248515

[B183] Xie S. Sukkar M. B. Issa R. Khorasani N. M. Chung K. F. (2007). Mechanisms of induction of airway smooth muscle hyperplasia by transforming growth factor-β. Am. J. Physiol. - Lung Cell. Mol. Physiol. 293 (1), L245–L253. 10.1152/ajplung.00068.2007 17468136 PMC1934553

[B184] Yang X. Lu Z. Wu H. Li W. Zheng L. Zhao J. (2018). “Collagen-alginate as bioink for three-dimensional (3D) cell printing based cartilage tissue engineering,” Mat. Sci. Eng. C 83, 195–201. 10.1016/j.msec.2017.09.002 29208279

[B185] Yadav P. Beniwal G. Saxena K. K. (2021). A review on pore and porosity in tissue engineering. Mat. Today Proc. 44, 2623–2628. 10.1016/j.matpr.2020.12.661

[B186] Yang J. Zhang Y. S. Yue K. Khademhosseini A. (2017). Cell-laden hydrogels for osteochondral and cartilage tissue engineering. Acta Biomater. 57, 1–25. 10.1016/j.actbio.2017.01.036 28088667 PMC5545789

[B187] Yong I. Oh S. W. Kim P. (2020). Re-engineered cell-derived extracellular matrix as a new approach to clarify the role of native ECM. Methods Cell. Biol. 156, 205–231. 10.1016/bs.mcb.2019.12.007 32222220

[B188] Yuan M. Pai P. J. Liu X. Lam H. Chan B. P. (2018). Proteomic analysis of nucleus pulposus cell-derived extracellular matrix niche and its effect on phenotypic alteration of dermal fibroblasts. Sci. Rep. 8 (1), 1512–1515. 10.1038/s41598-018-19931-9 29367647 PMC5784136

[B189] Zachman A. L. Crowder S. W. Ortiz O. Zienkiewicz K. J. Bronikowski C. M. Yu S. S. (2013). Pro-angiogenic and anti-inflammatory regulation by functional peptides loaded in polymeric implants for soft tissue regeneration. Tissue Eng. - Part A 19 (3–4), 437–447. 10.1089/ten.tea.2012.0158 22953721 PMC3542873

[B190] Zanotelli M. R. Goldblatt Z. E. Miller J. P. Bordeleau F. Li J. VanderBurgh J. A. (2018). Regulation of ATP utilization during metastatic cell migration by collagen architecture. Mol. Biol. Cell. 29 (1), 1–9. 10.1091/mbc.e17-01-0041 29118073 PMC5746062

[B191] Zeiger A. S. Loe F. C. Li R. Raghunath M. van Vliet K. J. (2012). Macromolecular crowding directs extracellular matrix organization and mesenchymal stem cell behavior. PLoS One 7 (5), e37904. 10.1371/journal.pone.0037904 22649562 PMC3359376

[B192] Zhang W. Liu Y. Zhang H. (2021a). Extracellular matrix: An important regulator of cell functions and skeletal muscle development. Cell. Biosci. 11 (1), 65–13. 10.1186/s13578-021-00579-4 33789727 PMC8011170

[B193] Zhang X. Guo W. G. Cui H. Liu H. Y. Zhang Y. Müller W. E. (2016). *In vitro* and *in vivo* enhancement of osteogenic capacity in a synthetic BMP-2 derived peptide-coated mineralized collagen composite. J. Tissue Eng. Regen. Med. 10 (2), 99–107. 10.1002/term.1705 23364810

[B194] Zhang W. Liu Y. Luo C. Zhai C. Li Z. Zhang Y. (2021b). Crosslinker-free silk/decellularized extracellular matrix porous bioink for 3D bioprinting-based cartilage tissue engineering. Mat. Sci. Eng. C 118, 111388. 10.1016/j.msec.2020.111388 33254994

[B195] Zhang Y. S. Khademhosseini A. (2015). Seeking the right context for evaluating nanomedicine: From tissue models in petri dishes to microfluidic organs-on-a-chip. Nanomedicine 10 (5), 685–688. 10.2217/nnm.15.18 25816872

[B196] Zhao Y. Feng B. Lee J. Lu N. Pierce D. M. (2019). A multi-layered model of human skin elucidates mechanisms of wrinkling in the forehead. J. Mech. Behav. Biomed. Mat. 105, 103694. 10.1016/j.jmbbm.2020.103694 32090898

[B197] Zhu W. Tringale K. R. Woller S. A. You S. Johnson S. Shen H. (2018). Rapid continuous 3D printing of customizable peripheral nerve guidance conduits. Mat. Today 21 (9), 951–959. 10.1016/j.mattod.2018.04.001 PMC6538503 31156331

[B198] Zimmermann D. R. Dours-Zimmermann M. T. Schubert M. Bruckner-Tuderman L. (1994). Versican is expressed in the proliferating zone in the epidermis and in association with the elastic network of the dermis. J. Cell. Biol. 124 (5), 817–825. 10.1083/jcb.124.5.817 8120102 PMC2119961

[B199] Zorlutuna P. Rong Z. Vadgama P. Hasirci V. (2009). Influence of nanopatterns on endothelial cell adhesion: Enhanced cell retention under shear stress. Acta Biomater. 5 (7), 2451–2459. 10.1016/j.actbio.2009.03.027 19394284

