# Supplementary material for: In vitro strategies for mimicking dynamic cell–ECM reciprocity in 3D culture models
Source: Front Bioeng Biotechnol. 2023 Jun 26;11:1197075. doi: 10.3389/fbioe.2023.1197075 (PMC10330728; doi:10.3389/fbioe.2023.1197075)
Supplement: Supplementary file 1 [file DataSheet1.PDF]

Table 1. ECM and GF derived peptides: examples, function and applications

**ECM-derived peptides**	*Applications and functions*
• DGEA (Asp-Gly-Glu-Ala)<br>• GFOGER (Gly-Phe-HPro-Gly-Glu-Arg)<br>• GFPGER (Gly-Phe-Pro-Gly-Glu-Arg)	Involved in integrin signaling that can promote cell adhesion, proliferation, and differentiation [19]
• PepGen P-15 (P-15): GTPGPQGIAGQRGVV (Gly-Thr-Pro-Gly-Pro-Gln-Gly-Ile-Ala-Gly-Gln-Arg-Gly-Val-Val)	Class of pro-adhesive collagen derived peptide It is known to stimulate osteoblast adhesion and proliferation [197].
• PHSRN (Pro-His-Ser-Arg-Asn)<br>• REDV (Arg-Glu-Asp-Val)<br>• LDV (Leu-Asp-Val)<br>• KQAGDV (Lys-Gln-Ala-Gly-Asp-Val)	Fibronectin derived sequences. Their use has been demonstrated to enhance adhesion and proliferation of fibroblasts, MSCs, and endothelial cells [19] [109].
• C16: KAFDITYVRLKF (Lys-Ala-Phe-Asp-Ile-Thr-Tyr-Val-Arg-Leu-Lys-Phe)	Other than pro-adhesive sequences, peptide from laminins has been found to have the pro-angiogenic feature. C16 can enhances endothelial cell migration, adhesion, and proliferation in vitro and it can support angiogenesis in vivo [110].
**GF-derived peptides**	
• P17: -IVAPPGYHAFYCHGECP (Ile-Val-Ala-Pro-Pro-Gly-Tyr-His-Ala-Phe-Tyr-Cys-His-Gly-Glu-Cys-Pro)-<br>• P24: -KIPKASSVPTELSAISTLYLSGGC (Lys-Ile-Pro-Lys-Ala-Ser-Ser-Val-Pro-Thr-Glu-Leu-Ser-Ala-Ile-Ser-Thr-Leu-Tyr-Leu-Ser-Gly-Gly-Cys)-<br>• BFP1: -GQGFSYPYKAVFSTQ (Gly-Gln-Gly-Phe-Ser-Tyr-Pro-Tyr-Lys-Ala-Val-Phe-Ser-Thr-Gln)-	BMP derived peptides. It has been shown to enhance viability of bone marrow stem cells, stimulate osteogenic differentiation and bone regeneration in combination with natural and synthetic scaffolds in vivo and in vitro [111]–[113].
• QK: -KLTWQELYQLKYKGI- (Lys-Leu-Thr-Trp-Gln-Glu-Leu-Tyr-Gln-Leu-Lys-Tyr-Lys-Gly-Ile)	Amino acid sequences that has been shown to mimic the VEGF functions by eliciting endothelial cell migrations, proliferation and angiogenesis [114].
